# PredictCBC-2.0: a contralateral breast cancer risk prediction model developed and validated in ~ 200,000 patients

**DOI:** 10.1186/s13058-022-01567-3

**Published:** 2022-10-21

**Authors:** Daniele Giardiello, Maartje J. Hooning, Michael Hauptmann, Renske Keeman, B. A. M. Heemskerk-Gerritsen, Heiko Becher, Carl Blomqvist, Stig E. Bojesen, Manjeet K. Bolla, Nicola J. Camp, Kamila Czene, Peter Devilee, Diana M. Eccles, Peter A. Fasching, Jonine D. Figueroa, Henrik Flyger, Montserrat García-Closas, Christopher A. Haiman, Ute Hamann, John L. Hopper, Anna Jakubowska, Floor E. Leeuwen, Annika Lindblom, Jan Lubiński, Sara Margolin, Maria Elena Martinez, Heli Nevanlinna, Ines Nevelsteen, Saskia Pelders, Paul D. P. Pharoah, Sabine Siesling, Melissa C. Southey, Annemieke H. van der Hout, Liselotte P. van Hest, Jenny Chang-Claude, Per Hall, Douglas F. Easton, Ewout W. Steyerberg, Marjanka K. Schmidt

**Affiliations:** 1 Division of Molecular Pathology, The Netherlands Cancer Institute - Antoni Van Leeuwenhoek Hospital, Plesmanlaan 121, 1066 CX Amsterdam, The Netherlands; 2 Department of Biomedical Data Sciences, Leiden University Medical Center, Leiden, The Netherlands; 3 Institute of Biomedicine, EURAC Research Affiliated Institute of the University of Lübeck, Bolzano, Italy; 4 Department of Medical Oncology, Erasmus MC Cancer Institute, Rotterdam, The Netherlands; 5 Brandenburg Medical School, Institute of Biostatistics and Registry Research, Neuruppin, Germany; 6 Institute of Medical Biometry and Epidemiology, University Medical Center Hamburg-Eppendorf, Hamburg, Germany; 7 Department of Oncology, Helsinki University Hospital, University of Helsinki, Helsinki, Finland; 8 Department of Oncology, Örebro University Hospital, Örebro, Sweden; 9 Copenhagen General Population Study, Herlev and Gentofte Hospital, Copenhagen University Hospital, Herlev, Denmark; 10 Department of Clinical Biochemistry, Herlev and Gentofte Hospital, Copenhagen University Hospital, Herlev, Denmark; 11 Faculty of Health and Medical Sciences, University of Copenhagen, Copenhagen, Denmark; 12 Department of Public Health and Primary Care, Centre for Cancer Genetic Epidemiology, University of Cambridge, Cambridge, UK; 13 Department of Internal Medicine and Huntsman Cancer Institute, University of Utah, Salt Lake City, UT USA; 14 Department of Medical Epidemiology and Biostatistics, Karolinska Institutet, Stockholm, Sweden; 15 Department of Pathology, Leiden University Medical Center, Leiden, The Netherlands; 16 Department of Human Genetics, Leiden University Medical Center, Leiden, The Netherlands; 17 Faculty of Medicine, University of Southampton, Southampton, UK; 18 Division of Hematology and Oncology, Department of Medicine, David Geffen School of Medicine, University of California at Los Angeles, Los Angeles, CA USA; 19 Department of Gynecology and Obstetrics, Comprehensive Cancer Center Erlangen-EMN, University Hospital Erlangen, Friedrich-Alexander University Erlangen-Nuremberg (FAU), Erlangen, Germany; 20 Usher Institute of Population Health Sciences and Informatics, The University of Edinburgh, Edinburgh, UK; 21 Cancer Research UK Edinburgh Centre, The University of Edinburgh, Edinburgh, UK; 22 Division of Cancer Epidemiology and Genetics, Department of Health and Human Services, National Cancer Institute, National Institutes of Health, Bethesda, MD USA; 23 Department of Breast Surgery, Herlev and Gentofte Hospital, Copenhagen University Hospital, Herlev, Denmark; 24 Department of Preventive Medicine, Keck School of Medicine, University of Southern California, Los Angeles, CA USA; 25 Molecular Genetics of Breast Cancer, German Cancer Research Center (DKFZ), Heidelberg, Germany; 26 Melbourne School of Population and Global Health, Centre for Epidemiology and Biostatistics, The University of Melbourne, Melbourne, VIC Australia; 27 Department of Genetics and Pathology, Pomeranian Medical University, Szczecin, Poland; 28 Independent Laboratory of Molecular Biology and Genetic Diagnostics, Pomeranian Medical University, Szczecin, Poland; 29 Division of Psychosocial Research and Epidemiology, The Netherlands Cancer Institute - Antoni Van Leeuwenhoek Hospital, Amsterdam, The Netherlands; 30 Department of Molecular Medicine and Surgery, Karolinska Institutet, Stockholm, Sweden; 31 Department of Clinical Genetics, Karolinska University Hospital, Stockholm, Sweden; 32 Department of Oncology, Södersjukhuset, Stockholm, Sweden; 33 Department of Clinical Science and Education, Karolinska Institutet, Södersjukhuset, Stockholm, Sweden; 34 Moores Cancer Center, University of California San Diego, La Jolla, CA USA; 35 Herbert Wertheim School of Public Health and Human Longevity Science, University of California San Diego, La Jolla, CA USA; 36 Department of Obstetrics and Gynecology, Helsinki University Hospital, University of Helsinki, Helsinki, Finland; 37 Department of Oncology, Leuven Multidisciplinary Breast Center, Leuven Cancer Institute, University Hospitals Leuven, Louven, Belgium; 38 Department of Oncology, Centre for Cancer Genetic Epidemiology, University of Cambridge, Cambridge, UK; 39 Department of Research and Development, Netherlands Comprehensive Cancer Organisation (IKNL), Utrecht, The Netherlands; 40 Department of HealthTechnology and Services Research, Technical Medical Centre, University of Twente, Enschede, The Netherlands; 41 Precision Medicine, School of Clinical Sciences at Monash Health, Monash University, Clayton, VIC Australia; 42 Department of Clinical Pathology, The University of Melbourne, Melbourne, VIC Australia; 43 Cancer Epidemiology Division, Cancer Council Victoria, Melbourne, VIC Australia; 44 Department of Genetics, University Medical Center Groningen, University Groningen, Groningen, The Netherlands; 45 Clinical Genetics, Amsterdam UMC, Vrije Universiteit Amsterdam, Amsterdam, The Netherlands; 46 Division of Cancer Epidemiology, German Cancer Research Center (DKFZ), Heidelberg, Germany; 47 Cancer Epidemiology Group, University Cancer Center Hamburg (UCCH), University Medical Center Hamburg-Eppendorf, Hamburg, Germany; 48 Department of Public Health, Erasmus MC Cancer Institute, Rotterdam, The Netherlands

**Keywords:** Contralateral breast cancer, Risk prediction, Contralateral preventive mastectomy, Clinical decision-making, Breast cancer genetic predisposition, Breast Cancer Association Consortium, BCAC, Prediction performance, *BRCA1/2* germline mutation, Polygenic risk score

## Abstract

**Background:**

Prediction of contralateral breast cancer (CBC) risk is challenging due to moderate performances of the known risk factors. We aimed to improve our previous risk prediction model (PredictCBC) by updated follow-up and including additional risk factors.

**Methods:**

We included data from 207,510 invasive breast cancer patients participating in 23 studies. In total, 8225 CBC events occurred over a median follow-up of 10.2 years. In addition to the previously included risk factors, PredictCBC-2.0 included *CHEK2* c.1100delC, a 313 variant polygenic risk score (PRS-313), body mass index (BMI), and parity. Fine and Gray regression was used to fit the model. Calibration and a time-dependent area under the curve (AUC) at 5 and 10 years were assessed to determine the performance of the models. Decision curve analysis was performed to evaluate the net benefit of PredictCBC-2.0 and previous PredictCBC models.

**Results:**

The discrimination of PredictCBC-2.0 at 10 years was higher than PredictCBC with an AUC of 0.65 (95% prediction intervals (PI) 0.56–0.74) versus 0.63 (95%PI 0.54–0.71). PredictCBC-2.0 was well calibrated with an observed/expected ratio at 10 years of 0.92 (95%PI 0.34–2.54). Decision curve analysis for contralateral preventive mastectomy (CPM) showed the potential clinical utility of PredictCBC-2.0 between thresholds of 4 and 12% 10-year CBC risk for *BRCA1/2* mutation carriers and non-carriers.

**Conclusions:**

Additional genetic information beyond *BRCA1/2* germline mutations improved CBC risk prediction and might help tailor clinical decision-making toward CPM or alternative preventive strategies. Identifying patients who benefit from CPM, especially in the general breast cancer population, remains challenging.

**Supplementary Information:**

The online version contains supplementary material available at 10.1186/s13058-022-01567-3.

## Introduction

Contralateral breast cancer (CBC) is the most common second primary cancer among women diagnosed with first primary invasive breast cancer (BC) [1]. CBC accounts for approximately 40–50% of all new secondary cancers in women with first primary invasive BC and has a potentially less favorable prognosis [2–6]. Worries regarding CBC risk have increased the demand for contralateral preventive mastectomy (CPM) [7, 8]. However, the impact of CPM on survival is uncertain, especially in women with a low risk to develop a CBC [9–13]. Thus, improved CBC risk prediction is important in order to inform decision-making on surveillance and preventive strategies. Currently, the most important factor for decision-making on CPM is the *BRCA1/2* mutation status [14].

We previously developed and cross-validated two models using data from 132,756 invasive BC patients with a median follow-up of 8.8 years including 4672 CBC events [15]. One model (PredictCBC-1A) was developed including information about *BRCA1/2* mutation status and another model (PredictCBC-1B) for the general breast cancer population of genetically untested women. Two other specific CBC prediction tools are currently available in the literature: the Manchester formula (part of the Manchester guidelines for CPM) and CBCrisk [15–18].

In addition to *BRCA1/2* mutations, other genetic risk factors for breast cancer are also associated with CBC risk. In particular, there is substantial evidence that the *CHEK2* c.1100delC variant increases the risk of developing CBC [19, 20]. In addition, polygenic risk scores (PRS) of common variants, developed for association with first breast cancer, have been shown to predict CBC in the general BC population and in *BRCA1/2* mutation carriers [21–24], particularly the extensively validated 313 SNP PRS [25]. With regard to the lifestyle and reproductive factors, there is evidence that body mass index (BMI) and parity at or around the time of the first primary invasive BC diagnosis are associated with CBC risk [26].

Our aim was to refit PredictCBC models incorporating these additional risk factors. We utilized the same dataset but with updated follow-up and added additional studies, especially one large study of *BRCA1* and *BRCA2* mutation carriers. We evaluated the potential improvement in prediction performance and utility for clinical decision-making of the updated models for both *BRCA1/2* carriers as the general (non-tested) breast cancer population (PredictCBC-2.0).

## Material and methods

### Study population and available data

We used the data from the same five main sources previously used for PredictCBC models to develop the PredictCBC-2.0 models including updated follow-up information, additional patients, and invasive or in situ CBC events [15]. Two studies were additionally included from the Breast Cancer Association Consortium (BCAC) compared to the version of the BCAC data used to develop PredictCBC-1A and PredictCBC-1B models. Most of the studies were either population- or hospital-based series; and most women were of European descent (Additional file 1: Data and patient selection and Additional file 2: Table S1 and Additional file 1: Table S2, available online). We also additionally included patients selected from the Hereditary Breast and Ovarian cancer study in the Netherlands (HEBON) [27], a nationwide study based on clinical genetic centers. The eligibility criteria were the same as previously: briefly, we included female patients with invasive first primary BC with no sign of distant metastases at diagnosis or prior history of any cancer (except for non-melanoma skin cancer) [15]. We included women diagnosed after 1990 so that diagnostic and treatment procedures were close to modern practice while follow-up was sufficient to study CBC incidence. In total, 207,510 women with first primary invasive BC from 23 studies were included. All studies were approved by the appropriate ethics and scientific review boards. All women provided written informed consent; or, for some Dutch cohorts as applicable, the secondary use of clinical data was in accordance with Dutch legislation and codes of conduct [28, 29]. Information about the sample size for every data source and the total sample size after eligibility criteria are provided in Table 1. The choice of additional predictors in the analyses was based on evidence from the literature and the availability of predictors in our data sources. In particular, evidence from the literature suggests that *CHEK2 c.1100delC* and 313 SNP PRS increased the risk of developing CBC [21–24]. In addition, a systematic review of lifestyle and reproductive factors suggested that BMI and parity at or around the time of the first primary invasive BC diagnosis are associated with CBC risk [26]. Details about sample size per study and about the factors included in the analyses, follow-up per dataset, and study design are in Additional file 2: Table S1 and Additional file 3: Table S3, available online.

**Table 1:** Patient characteristics in the different data sources

	Source of data					
	ABCS	BCAC^‡^	BOSOM	EMC	HEBON	NCR
Number of patients	2763	186,594	7105	3483	16,617	160,861
Eligibility criteria, *number of patients excluded*						
Studies from Asian countries	–	7146	–	–	–	–
Patients of non-European descent	74	51,328	–	–	–	–
Patients younger than 18 years old	–	4	–	–	–	–
Year of PBC diagnosis before 1990	–	4014	3126	–	1132	–
Year of PBC diagnosis missing	–	15,435	–	–	2	–
PBC stage 0	123	38	2	–	–	–
PBC stage IV	149	1811	104	–	115	7774
Patients did not undergo surgery	24	1247	43	5	293	9278
Number of eligible patients	2393	105,571	3830	3478	15,075	143,809
No follow-up or follow-up less than 3 months	173	15,804	70	88	2382*	3396
Familiar breast cancer studies	–	6739	–	–	–	–
Studies with less than 10 CBC events	–	37,994	–	–	–	–
Number of patients included in the analysis (number of patients with CBC)	2220 (44)	45,034 (1001)	3760 (288)	3390 (221)	12,693 (918)	140,413 (5753)
Total number of patients included in the analysis (number of CBC)	207,510 (8225 of which 6828 invasive and 1397 in situ)					

*ABCS*: Amsterdam Breast Cancer Study, *BCAC* Breast Cancer Association Consortium, *BOSOM* Breast Cancer Outcome Study of Mutation carriers, *EMC* Erasmus Medical Center, *HEBON* Hereditary Breast and Ovarian cancer study Netherlands, *NCR* Netherlands Cancer Registry, *PBC* primary breast cancer, *CBC* contralateral breast cancer

^*^1433 tested for *BRCA1/2* germline mutation after CBC or preventive mastectomy

^‡^BCAC is composed of 106 studies worldwide. The 45,034 patients selected for the analysis came from 18 studies

### Statistical analyses

#### Primary endpoint and follow-up

The primary endpoint in the analyses was the incidence of invasive or in situ metachronous CBC. Follow-up started 3 months after invasive first primary BC diagnosis, to exclude synchronous CBCs, and ended at the date of CBC, distant metastasis (but not a loco-regional relapse), CPM, or last date of follow-up (due to death, loss to follow-up, or end of study), whichever occurred first. For 36,553 (17.6%) women, from BCAC and HEBON, recruitment or blood sampling for DNA testing occurred more than 3 months after diagnosis of the first primary BC. For women with the first primary invasive BC, follow-up started at recruitment or at the date of blood draw or at DNA test result (left truncation). Patients who underwent CPM during the follow-up were censored because of negligible CBC risk after a CPM [30]. Missing data were multiply imputed by chained equations (MICE) to avoid loss of information due to case-wise deletion [31–33] (Additional file 1: Multiple imputation of missing values, available online).

#### Model development and validation

We used multivariable Fine and Gray regression models to account for death and distant metastases as competing events [34]. Analyses were stratified by a study to allow baseline hazard (sub)distributions to differ across studies. The assumption of proportional subdistribution hazards was graphically checked using Schoenfeld residuals [35]. The resulting subdistribution hazard ratios (sHRs) and corresponding 95% confidence intervals (CI) were pooled from 5 imputed datasets using Rubin’s rules [33]. We re-estimated the coefficients of PredictCBC-1A and PredictCBC-1B, and we re-fitted the PredictCBC models using the extended dataset with updated follow-up time. PredictCBC-1A, developed including information about *BRCA1/2* mutation carrier status, was extended by including *CHEK2* c.1110delC status, PRS-313, self-reported BMI, and self-reported parity (hereafter: PredictCBC-2.0A) [15]. *CHEK2* c.1110delC and PRS-313 were derived from the BCAC database, as published previously [25, 36, 37]. We extended PredictCBC-1B, developed for genetically untested women, incorporating self-reported BMI and parity (hereafter: PredictCBC-2.0B). Potential nonlinear relations between continuous predictors and CBC risk were investigated using restricted cubic splines with three knots.

The validity of the model was investigated by leave-one-study-out cross-validation [38]. In each validation cycle, all studies were analyzed except one, in which the validity of the model was evaluated. Since some BCAC studies had insufficient CBC events required for reliable validation, we used the geographic area as a unit for splitting [38–40]. Nineteen out of 23 studies were combined in 4 geographic areas (Additional file 1: Table S2, available online). A total of 8 units of splitting including 4 geographic areas and 4 studies were used to cross-validate the models.

The performance of the PredictCBC-2.0 was assessed by discrimination, i.e., the ability to differentiate between patients diagnosed with CBC and those who were not, and by calibration, which measures the agreement between the actual (observed) risk and CBC risk estimated by the prediction models (predicted). Discrimination was quantified by time-dependent areas under the ROC curve (AUCs) based on Inverse Censoring Probability Weighting at 5 and 10 years [41]. The AUCs were estimated using the prognostic index which is a/the combination of the estimated coefficients (betas) of PredictCBC models multiplied by the corresponding individual characteristics (i.e., predictors) included in the models. Values of AUCs close to 1 indicate good discrimination, while values close to 0.5 indicated poor discrimination. Calibration was assessed by the observed-to-expected (O/E) ratio and calibration plots at 5 and 10 years [42, 43]. An O/E ratio lower or higher than 1 indicates that average predictions are too high or low, respectively.

To consider heterogeneity among studies, a random-effect meta-analysis was performed to provide summaries of discrimination and calibration performance. The 95% prediction intervals (PI) indicate the likely performance of the model in a new dataset. The summary performances of PredictCBC-2.0 and 1.0 models were compared to evaluate whether adding the new predictors improved the performance of CBC risk prediction. We developed and validated the risk prediction model following the Transparent Reporting of a Multivariable Prediction model for Individual Prognosis or Diagnosis (TRIPOD) statement [44]. Analyses were done in SAS (SAS Institute Inc., Cary, NC, USA) and R (version 3.6.1).

### Clinical utility

The clinical utility of the prediction models was evaluated using decision curve analysis (DCA) [45, 46]. A key metric DCA is the net benefit, which is the number of true-positive classifications (in this example: the number of CPMs in patients who would have developed a CBC) minus the weighted number of false-positive classifications (in this example: the number of unnecessary CPMs in patients who would not have developed a CBC). The false positives are weighted by a factor related to the relative harm of a missed CBC versus an unnecessary CPM. The weighting is derived from the threshold probability to develop a CBC using a fixed time horizon (e.g., CBC risk at 5 or 10 years) [47]. For example, a threshold of 10% implies that CPM in 10 patients, of whom one would develop CBC if untreated, is acceptable (thus performing 9 unnecessary CPMs). The net benefit of a prediction model is traditionally compared with the strategies of treat all or treat none. Since the use of CPM is generally only considered among *BRCA1/2* mutation carriers, the decision curve analysis was reported among *BRCA1/2* mutation carriers and non-carriers separately [48]. Among patients not tested for *BRCA1/2* germline mutations, we assumed that the decision for CPM is based on family history of breast cancer. The net benefits of PredictCBC-2.0A and PredictCBC-2.0B were compared with the net benefit of PredictCBC-1A and 1B, respectively, to assess the potential improvement in the clinical utility of the updated models.

## Results

A total of 207,510 women with invasive first primary BC diagnosed between 1990 and 2017, with 8225 CBC events (6828 invasive, 1397 in situ), from 23 studies, were used for CBC risk prediction modeling (Additional file 2: Table S1, available online). Median follow-up time was 10.2 years, and CBC cumulative incidences at 5 and 10 years were 2.2% and 4.1%, respectively. Details of the studies and patient, tumor, and treatment characteristics are provided in Additional file 3: Table S3 (available online). The multivariable models with estimates for all included factors are given in Table 2.

**Table 2:** Multivariable subdistribution hazard models for contralateral breast cancer risk

Factor (reference)	PredictCBC-2.0A	PredictCBC-2.0B
	sHR (95% CI)	sHR (95% CI)
Age at PBC, *years* (75th vs. 25th quartile: 66 vs. 48)	0.87^a^ (0.83–0.90)	0.82^a^ (0.78–0.85)
Body mass index, *kg/m*^*2*^ (75th vs. 25th quartile: 28.4 vs. 22.7)	1.06 (1.03–1.09)	1.06 (1.03–1.09)
Parity (75th vs. 25th quartile: 3 vs. 1)	0.85 (0.82–0.88)	0.86 (0.83–0.90)
First-degree family history of BC (yes)	1.17 (1.12–1.23)	1.35 (1.29–1.42)
*BRCA mutation*		
*BRCA1* versus non-carrier	4.79 (4.43–5.17)	–
*BRCA2* versus non-carrier	3.09 (2.72–4.25)	–
PRS_313_^b^ (75th vs. 25th quartile: -0.49 vs. 0.32)	1.35 (1.31–1.39)	–
*CHEK2* c.1100delC mutation (present)	2.75 (2.85–3.34)	–
Nodal status of PBC (positive)	0.99 (0.93–1.05)	0.99 (0.93–1.04)
*Tumor size category of PBC, cm*		
(2,5] versus ≤ 2	0.99 (0.94–1.05)	1.01 (0.96–1.07)
> 5 versus ≤ 2	1.23 (1.10–1.36)	1.22 (1.09–1.36)
Morphology of PBC (lobular including mixed)	1.19 (1.12–1.27)	1.17 (1.10–1.24)
*Grade of PBC*		
Moderately differentiated vs. well differentiated (II vs. I)	0.93 (0.88–0.99)	0.98 (0.93–1.04)
Poorly differentiated vs. well differentiated (III vs. I)	0.85 (0.79–0.91)	0.95 (0.88–1.01)
Chemotherapy (yes)	0.75 (0.70–0.80)	0.75 (0.70–0.80)
Radiotherapy to the breast (yes)	0.93 (0.89–0.98)	0.95 (0.90–0.99)
*ER with endocrine therapy*		
Negative/no versus positive/yes	1.53 (1.43–1.65)	1.78 (1.67–1.90)
Positive/no versus positive/yes	1.95 (1.83–2.07)	1.94 (1.82–2.06)
HER2 with trastuzumab therapy		
Negative/no versus positive/yes	1.22 (1.09–1.38)	1.30 (1.15–1.46)
Positive/no versus positive/yes	1.12 (0.97–1.28)	1.14 (1.00–1.31)

*vs.* versus, *sHR* subdistributional hazard ratio, *CI* confidence interval, *PRS* polygenic risk score, *BC* breast cancer, *PBC* first primary breast cancer, *ER* estrogen receptor, *HER2* human epidermal growth factor 2

^a^age was parameterized as a linear spline with one interior knot at 60 years. For representation purposes, we here provide the sHR for the 75th versus the 25th percentile

^b^PRS standardized by the same standard deviation (SD) used by Mavaddat et al. (SD = 0.61)[25]

Most of the factors were independently associated with CBC risk, including the new factors incorporated in the PredictCBC-2.0 models, i.e., s BMI, parity, *CHEK2* c.1110delC, and PRS-313. There was no evidence against log-linear relationships between BMI, parity and PRS-313 and CBC risk. Nonlinearity between age at first BC diagnosis and CBC risk was accounted for with a linear spline at age 60 years. The formulae of the PredictCBC models are provided in Additional file 1: Formula to estimate the contralateral breast cancer risk using PredictCBC-2.0A and PredictCBC-2.0B (available online). To calculate the predicted CBC cumulative incidence, we used the event-free baseline probability of the Netherlands Cancer Registry (NCR), as previously [15].

**Fig. 1:**
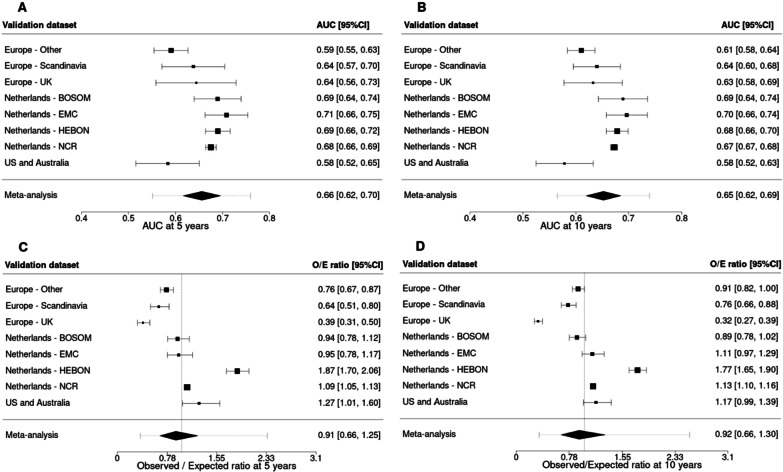
Analysis of predictive performance of PredictCBC-2.0A in leave-one-study-out cross-validation. Discrimination was assessed by a time-dependent AUC at 5 and 10 years (panel **A** and **B**, respectively). Calibration accuracy was measured with observed/expected (O/E) ratio at 5 and 10 years (panel **C** and **D**, respectively). The black squares indicate the estimated accuracy of a model built using all remaining studies or geographic areas. The black horizontal lines indicate the corresponding 95% confidence intervals of the estimated accuracy (interval whiskers). The black diamonds indicate the mean with the corresponding 95% confidence intervals of the predictive accuracy, and the dashed horizontal lines indicate the corresponding 95% prediction intervals

**Fig. 2:**
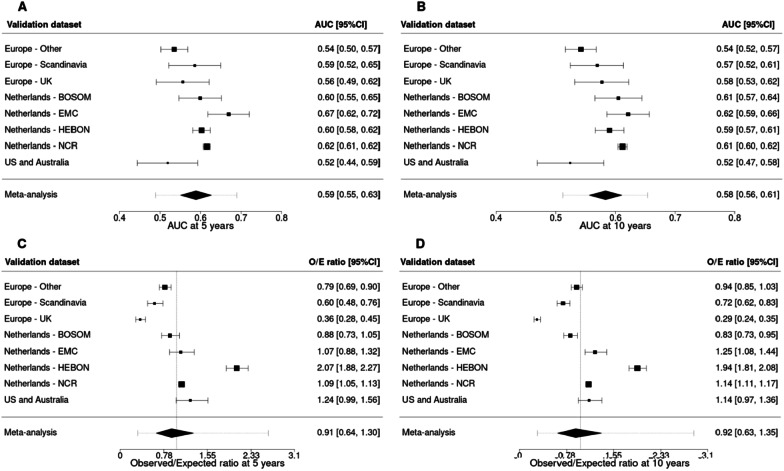
Analysis of predictive performance of PredictCBC-2.0B in leave-one-study-out cross-validation. Discrimination was assessed by a time-dependent AUC at 5 and 10 years (panel **A** and **B**, respectively). Calibration accuracy was measured with observed/expected (O/E) ratio at 5 and 10 years (panel **C** and **D**, respectively). The black squares indicate the estimated accuracy of a model built using all remaining studies or geographic areas. The black horizontal lines indicate the corresponding 95% confidence intervals of the estimated accuracy (interval whiskers). The black diamonds indicate the mean with the corresponding 95% confidence intervals of the predictive accuracy, and the dashed horizontal lines indicate the corresponding 95% prediction intervals

The AUCs at 5 and 10 years of PredictCBC-2.0A were higher than of PredictCBC-1A at 5 years: 0.66, 95% prediction interval (PI) 0.55–0.76 versus 0.62 (95%PI 0.51–0.74); and at 10 years: 0.65 (95%PI 0.56–0.74) versus 0.63 (95%PI 0.54–0.71) (Figs. 1 and 2, Table 3). The AUCs for PredictCBC-2.0B and PredictCBC-1B were both 0.59 (95%PI: PredictCBC-2.0B: 0.51–0.68; PredictCBC-1B:0.49–0.69) at 5 years and both 0.58 (95%PI 0.51–0.65) at 10 years (Figs. 1 and 2, Table 3).

**Table 3:** Summary of prediction performance of PredictCBC-1A, PredictCBC-1B, PredictCBC-2.0A, and PredictCBC-2.0B with the corresponding 95% prediction intervals (PI) based on a leave-one-study-out cross-validation procedure

CBC risk prediction model	Performance measure			
	Discrimination		Calibration	
	AUC (95% PI)		O/E ratio (95% PI)	
	5-year	10-year	5-year	10-year
PredictCBC-1A	0.62 (0.51–0.74)	0.63 (0.54–0.71)	0.90 (0.36–2.24)	0.91 (0.34–2.48)
PredictCBC-2.0A	0.66 (0.55–0.76)	0.65 (0.56–0.74)	0.91 (0.35–2.34)	0.92 (0.34–2.54)
PredictCBC-1B	0.59 (0.49–0.69)	0.58 (0.51–0.65)	0.91 (0.32–2.55)	0.92 (0.30–2.80)
PredictCBC-2.0B	0.59 (0.51–0.68)	0.58 (0.51–0.65)	0.91 (0.31–2.63)	0.92 (0.30–2.87)

*AUC* area under the curve, *CBC* contralateral breast cancer, *PI* prediction interval, *O/E* observed/expected

The O/E ratio at 5 and 10 years across all versions of PredictCBC models ranged between 0.90 and 0.92 with similar 95%PIs (Figs. 1 and 2, Table 3). Calibration plots of PredictCBC-2.0 models are provided in Additional file 1: Figs, S1–S4 (available online).

**Fig. 3:**
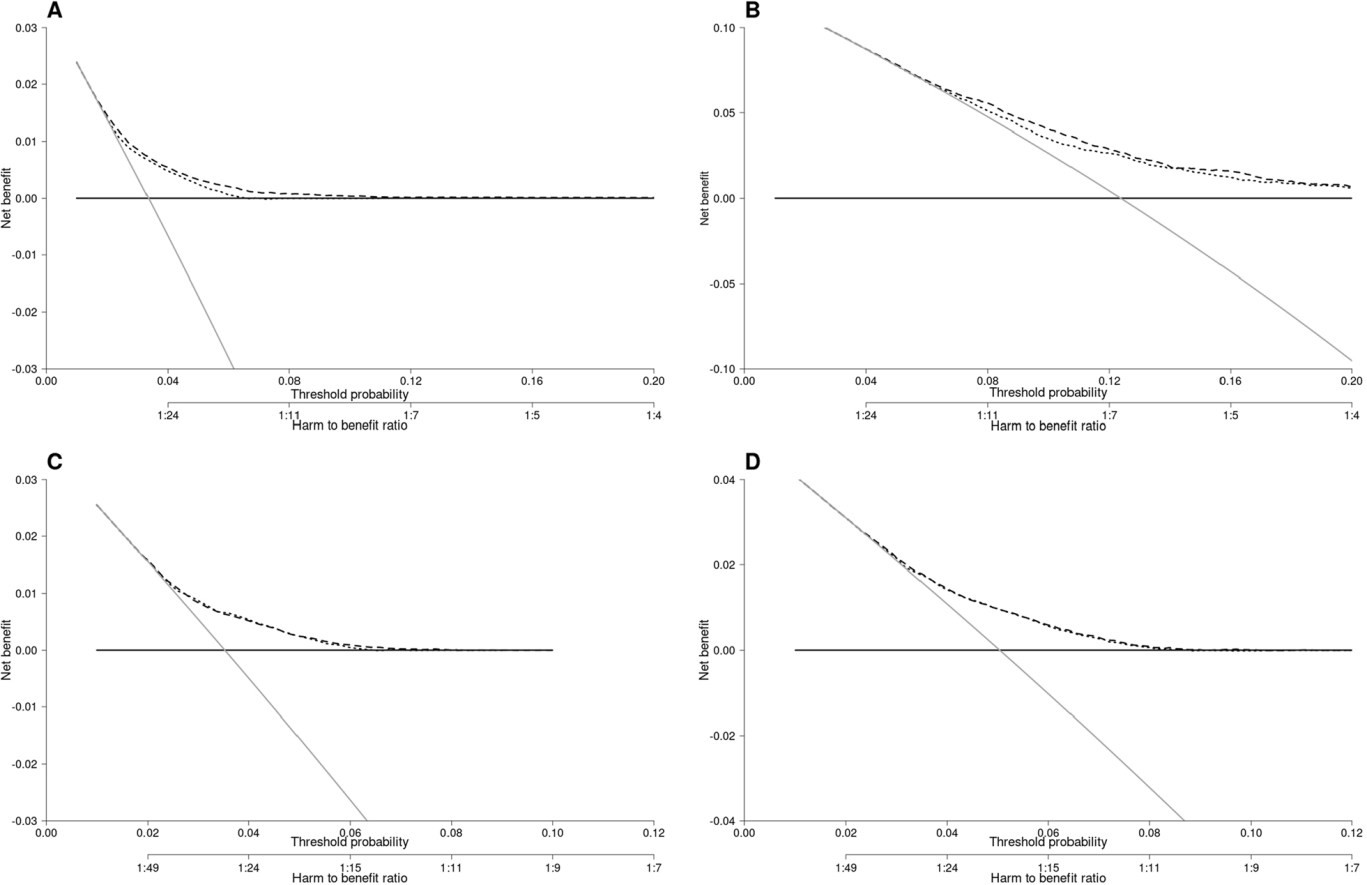
Decision curve analysis at 10 years for the contralateral breast cancer risk (CBC) models (PredictCBC-1.0 and PredictCBC-2.0 models) including *BRCA* mutation information. **A** The decision curve to determine the net benefit of the estimated 10-year predicted CBC cumulative incidence for patients without a *BRCA1/2* gene mutation using PredictCBC-1A (dotted black line) and PredictCBC-2.0A (dashed black line) compared to not treating any patients with contralateral preventive mastectomy (CPM) (black solid line). **B** The decision curve to determine the net benefit of the estimated 10-year predicted CBC cumulative incidence for *BRCA1/2* mutation carriers using PredictCBC-1A (dotted black line), PredictCBC-2.0A (dashed black line) versus treating (or at least counseling) all patients (gray solid line). **C** The decision curve to determine the net benefit of the estimated 10-year predicted CBC cumulative incidence for patients without (first degree) family history using PredictCBC-1B (dotted black line), PredictCBC-2.0B (dashed black line) compared to not treating any patients with CPM (black solid line). **D** The decision curve to determine the net benefit of the estimated 10-year predicted CBC cumulative incidence for patients with (first degree) family history using PredictCBC-1B (dotted black line), PredictCBC-2.0B (dashed black line) versus treating (or at least counseling) all patients (gray solid line). The y-axis measures net benefit, which is calculated by summing the benefits (true positives, i.e., patients with a CBC who needed a CPM) and subtracting the harms (false positives, i.e., patients with CPM who do not need it). The latter are weighted by a factor related to the relative harm of a non-prevented CBC versus an unnecessary CPM. The factor is derived from the threshold probability to develop a CBC at 10 years at which a patient would opt for CPM (e.g., 10%). The x-axis represents the threshold probability. Using a threshold probability of 10% implicitly means that CPM in 10 patients of whom one would develop a CBC if untreated is acceptable (9 unnecessary CPMs, harm-to-benefit ratio 1:9)

**Fig. 4:**
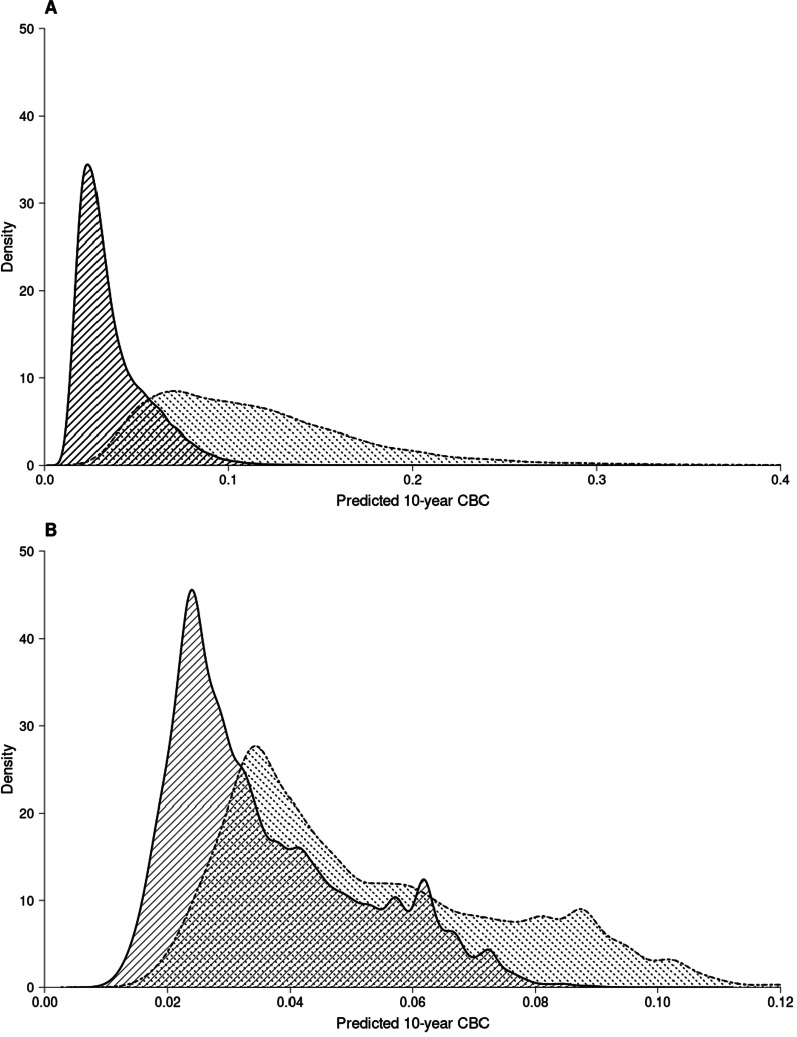
Density distribution of 10-year predicted contralateral breast cancer using PredictCBC version 2 models. **A** Density distribution of 10-year predicted contralateral breast cancer absolute risk using PredictCBC-2.0A within non-carriers (area with black solid lines) and *BRCA1/2* mutation carriers (area with black dashed lines). **B** Density distribution of 10-year predicted contralateral breast cancer absolute risk using PredictCBC-2.0B within patients without (first degree) family history (area with black solid lines) and patients with (first degree) family history (area with black dashed lines)

The decision curves showed the net benefit for a range of harm–benefit thresholds at 10-year CBC risk (Fig. 3). We evaluated the potential clinical utility of PredictCBC-2A versus PredictCBC-1.0A for decision thresholds between 4 and 12% for the 10-year CBC risk among *BRCA1/2* mutation carriers and non-carriers (Figs. 3 and 4, Table 4). For example, if consensus guidelines would indicate the acceptability of 1 in 10 patients for whom a CPM is recommended developing CBC, a risk threshold of 10% may be used to define high- and low-risk *BRCA1/2* mutation carriers based on the absolute 10-year CBC risk prediction estimated by the models. Compared with a strategy recommending CPM to all *BRCA1*/2 mutation carriers, PredictCBC-1A avoids 76.9 net CPMs per 1000 patients (Table 4). An additional 50.0 CPMs may be avoided using PredictCBC-2.0A compared to PredictCBC-1A. In contrast, almost no non-*BRCA1/2* mutation carriers had predictions above the 10% threshold (general BC population, Table 4); three necessary CPMs per 1000 patients would be indicated using PredictCBC-2.0A. Analyses for PredictCBC-1B and PredictCBC-2.0B at 10 years suggested a potential clinical utility between 4 and 6% 10-year CBC risk for patients with and without family history (Table 4 and Figs. 3 and 4). No remarkable improvement in net benefit was detected using PredictCBC-2.0B compared to PredictCBC-1B in decision-making regarding CPM (Table 4 and Fig. 3). Decision curves for CBC risk using PredictCBC and PredictCBC-2.0 at 5 years and the corresponding clinical utility showed similar patterns (Additional file 1: Figs. S5-S6 and Table S4, available online).

**Table 4:** Clinical utility of the 10-year contralateral breast cancer risk prediction models (PredictCBC-1A with PredictCBC-2.0A and PredictCBC-1B with PredictCBC-2.0B)

*PredictCBC-1A and PredictCBC-2.0A*							
Probability threshold *p*_*t*_ (%)	Unnecessary CPMs needed to detect one necessary CPM*	*BRCA1/2* mutation carriers			Non-carriers		
		Net benefit versus treat all patients with CPM (per 1000)	Avoided unnecessary CPMs per 1000 patients using PredictCBC-1A	Additional avoided unnecessary CPMs per 1000 patients using PredictCBC-2.0A	Net benefit versus treat none (per 1000)	Performed necessary CPMs per 1000 patients using PredictCBC-1A	Additional performed necessary CPMs per 1000 patients using PredictCBC-2.0A
4	24	0.1	0.3	1.9	4.8	115.7	15.3
6	15.7	No benefit	0.0	20.0	0.6	9.3	22.9
8	11.5	3.5	40.6	52.0	No benefit	0.0	9.0
10	9.0	8.5	76.9	50.2	No benefit	0.0	3.4
12	7.3	22.4	164.0	15.0	No benefit	0.0	1.1

*PredictCBC-1B and PredictCBC-2.0B*							
Probability threshold *p*_*t*_ (%)	Unnecessary CPMs needed to detect one necessary CPM*	Family history			No family history		
		Net benefit versus treat all patients with CPM (per 1000)	Avoided unnecessary CPMs per 1000 patients using PredictCBC-1B	Additional avoided unnecessary CPMs per 1000 patients using PredictCBC-2.0B	Net benefit versus treat none (per 1000)	Performed necessary CPMs per 1000 patients using PredictCBC-1B	Additional performed necessary CPMs per 1000 patients using PredictCBC-2.0B
4	24	3.4	80.8	5.9	5.4	130.4	0.0
5	19	9.4	177.9	0.0	2.4	46.5	0.1
6	15.7	15.9	248.7	4.0	0.5	7.1	7.5

For PredictCBC versions 1A and 2.0A, at the same probability threshold, the net benefit is exemplified in *BRCA1/2* mutation carriers (for avoiding unnecessary CPM) and non-carriers (performing necessary CPM). For PredictCBC versions 1B and 2.0B, at the same probability threshold, the net benefit is exemplified in patients with family history (for avoiding unnecessary CPM) and patients without family history (performing necessary CPM)

*CPM* contralateral preventive mastectomy

^*^The number of unnecessary contralateral mastectomies needed to detect one necessary CPM is calculated by: (1 − *p*_*t*_)/*p*_*t*_

## Discussion

We evaluated the potential improvement in CBC risk prediction by adding established genetic (*CHEK2* c.1100delC and PRS-313) and lifestyle (BMI and parity) factors to the previous PredictCBC models and used additional follow-up information and new studies to provide more reliable estimates.

The current clinical recommendations of CPM are mostly based on the presence of a pathogenic mutation in *BRCA1/2* [49, 50]. This seems a reasonable approach according to CBC risk predictions based on the PredictCBC models: few non-*BRCA1/2* carriers exceed a 10% 10-year risk threshold. However, approximately 40% of *BRCA1/2* mutation carriers do not reach this threshold either, suggesting that a significant proportion of *BRCA1/2* carriers might be spared CPM. Additional genetic information beyond *BRCA1/2* germline mutation such as the presence of the *CHEK2* c.1110delC variant and PRS-313 might improve decision-making.

Currently available CBC models, such as CBCrisk and the Manchester formula, show only moderate discrimination [51]. In addition, the Manchester formula has been shown to systematically overestimate CBC risk [51]. The BOADICEA model, a well-known risk prediction tool to estimate the risk of developing the first primary BC, also allows the calculation of CBC risk [52–55]. Although BOADICEA includes rare pathogenic variants in moderate- and high-risk BC susceptibility genes (i.e., *BRCA1*, *BRCA2*, *PALB2*, *ATM* and *CHEK2*, *BARD1*, *RAD51C*, *RAD51D*), and PRS-313, it does not incorporate information on the systemic treatment of the primary BC, which are important predictors of CBC risk [56].

A model for the prediction of recurrence, the INFLUENCE nomogram, was developed to estimate 5-year recurrence risk as well as conditional annual risks of developing a local or regional recurrence based on first BC and treatment characteristics [57]. A more recent version (INFLUENCE 2.0) also provides 5-year individualized predictions for secondary primary breast cancer based on cases older than 50 years at first cancer diagnosis from the NCR nationwide cohort irrespective of their genetic status or testing status using random survival forests [58]. The model provided moderate discrimination (AUC at 5 years: 0.67; 95%CI 0.65–0.68) using internal validation. In our comparable population- and hospital-based Dutch series, EMC and NCR, the AUCs at 5 years of PredictCBC-1A were 0.69 (95%CI 0.64–0.73) and 0.66 (95%CI 0.65–0.67), and of PredictCBC-2.0A 0.71 (95%CI 0.66–0.75) and 0.68 (95%CI 0.66–0.69), respectively. Moreover, INFLUENCE 2.0 is only relevant to the general population, while PredictCBC can also be used in the clinical genetic setting. Notably, we demonstrated that decision-making about preventive strategies in clinical practice is unlikely to improve without genetic information.

Our work has some limitations: firstly, some women included in the Dutch studies (providing specific information on family history, *BRCA* mutation or CPM) were also present in our selection of the NCR population, as described previously [15]. Privacy and coding issues prevented linkage at the individual patient level, but based on the hospitals from which the studies were recruited, and the age and period criteria used, we calculated a maximum potential overlap of 9%. Secondly, important predictors such as family history, *BRCA1/2* and *CHEK2* c.1110delC status, and PRS-313, were only available in a subset of the women, although the multiple imputation approach should lead to consistent estimates [59–61]. Detailed information about family history of breast cancer would have been useful to improve CBC risk prediction, especially among patients with a mutation in *BRCA1/2* or *CHEK2*. Nonetheless, we considerably increased the number of patients with *BRCA1/2* mutation status and family history information compared to our previous publication (40,343 vs. 7704 and 53,399 vs. 30,541 patients with available *BRCA* mutation status and family history information, respectively), and added *CHEK2* c.1110delC, which is a founder mutation present in approximately 0.5–1.6% of individuals of Northern and Eastern European descent and explains the large majority of carriers of *CHEK2* protein truncating variants in these populations [19, 62]. Further validation will be required to investigate how well PredictCBC models predict risk in other populations. In particular, the model was developed in patients of European ancestry and further evaluation and adaptation will be needed to extend PredictCBC models to non-European populations, including Asia [63, 64]. Future research might also include comparisons of machine learning (ML) methods with classical statistical regression models [65, 66].

The prediction models may be further improved by including additional risk factors. In particular, rare mutations in other breast cancer susceptibility genes, such as *ATM* and *PALB2*, are also likely to be associated with an increased risk of CBC [22, 67, 68]. The discrimination provided by the PRS will also improve as more SNPs are added [69, 70]. Prediction performance might also be improved by adding breast density and other risk factors (e.g., additional lifestyle and reproductive factors such as alcohol use, age at primiparity, age at menopause) modeled dynamically in a time-dependent fashion [71]. Finally, we wish to emphasize that adequate presentation (e.g., with online tools) of the risk estimates is crucial for effective communication about CBC risk during doctor–patient consultations [72, 73].

## Conclusions

In conclusion, we present an updated version of a previously proposed contralateral breast cancer risk model (PredictCBC) including additional information on breast cancer genetic variants beyond *BRCA1/2*, lifestyle and reproductive factors. PredictCBC-2.0, available online at [74], is based on longer follow-up from a wide range of new European-descent population and hospital-based studies, with reasonable calibration. PredictCBC-2.0 may be used to tailor clinical decision-making toward CPM or alternative preventive strategies, especially when genetic information is available.

## Supplementary Information

**Additional file 1.** Supplementary methods also including the following tables and figures **Table S2**. List of BCAC studies (including ABCS source) with the corresponding country and geographic area. Table S4: Clinical utility of the 5-year contralateral breast cancer risk prediction models (PredictCBC-1A with PredictCBC-2.0A and PredictCBC-1B with PredictCBC-2.0B). **Figure S1.** Visual assessment of calibration through calibration plots in the internal–external cross-validation at 5 years for the PredictCBC-2.0A model. **Figure S2.** Visual assessment of calibration through calibration plots in the internal–external cross-validation at 10 years for the PredictCBC-2.0A model. **Figure S3.** Visual assessment of calibration through calibration plots in the internal–external cross-validation at 5 years for the PredictCBC-2.0B model. **Figure S4.** Visual assessment of calibration through calibration plots in the internal–external cross-validation at 10 years for the PredictCBC-2.0B model. **Figure S5.** Density distribution of 5-year predicted contralateral breast cancer using PredictCBC-2.0 models. **Figure S6.** Decision curve analysis at 5 years for the contralateral breast cancer risk models (PredictCBC and PredictCBC-2.0) including *BRCA* mutation information.

**Additional file 2: Table S1.** Description of the studies included in the analyses.

**Additional file 1: Table S3.** Patient and primary breast cancer characteristics per study.

## Data Availability

The datasets analyzed during the current study are not publicly available due to the protection of participant privacy and confidentiality, and ownership of the contributing institutions, but may be made available in an anonymized form via the corresponding author on reasonable request and after approval of the involved institutions.

## Abbreviations

AUC: Area under the ROC curve
BC: Breast cancer
BCAC: Breast Cancer Association Consortium
BMI: Body mass index
CBC: Contralateral breast cancer
CI: Confidence interval
CPM: Contralateral preventive mastectomy
DCA: Decision curve analysis
ER: Estrogen receptor
HEBON: The Hereditary Breast and Ovarian Cancer Research Group Netherlands
HER2: Human epidermal growth receptor 2
MICE: Multiple imputation by chained equations
O/E: Observed/expected
NCR: Netherlands Cancer Registry
PI: Prediction interval
PR: Progesterone receptor
PRS: Polygenic risk score
sHR: Subdistribution hazard ratio
TRIPOD: Transparent reporting of a multivariable prediction model for individual prognosis or diagnosis

## References

[1] Chen Y, Thompson W, Semenciw R, Mao Y (1999). Epidemiology of contralateral breast cancer. Cancer Epidemiol Biomarkers Prev.

[2] Gao X, Fisher SG, Emami B (2003). Risk of second primary cancer in the contralateral breast in women treated for early-stage breast cancer: a population-based study. Int J Radiat Oncol Biol Phys.

[3] Curtis RE, Ron E, Hankey BF, Hoover RN. New malignancies following breast cancer. In: New malignancies among cancer survivors: SEER Cancer Registries, 1973–2000; 181–205.

[4] Yu GP, Schantz SP, Neugut AI, Zhang ZF (2006). Incidences and trends of second cancers in female breast cancer patients: a fixed inception cohort-based analysis (United States). Cancer Causes Control.

[5] Soerjomataram I, Louwman WJ, Lemmens VE, de Vries E, Klokman WJ, Coebergh JW (2005). Risks of second primary breast and urogenital cancer following female breast cancer in the south of The Netherlands, 1972–2001. Eur J Cancer.

[6] Schaapveld M, Visser O, Louwman WJ, Willemse PH, de Vries EG, van der Graaf WT, Otter R, Coebergh JW, van Leeuwen FE (2008). The impact of adjuvant therapy on contralateral breast cancer risk and the prognostic significance of contralateral breast cancer: a population based study in the Netherlands. Breast Cancer Res Treat.

[7] Tuttle TM, Habermann EB, Grund EH, Morris TJ, Virnig BA (2007). Increasing use of contralateral prophylactic mastectomy for breast cancer patients: a trend toward more aggressive surgical treatment. J Clin Oncol.

[8] Narod SA (2014). Bilateral breast cancers. Nat Rev Clin Oncol.

[9] Metcalfe K, Gershman S, Ghadirian P, Lynch HT, Snyder C, Tung N, Kim-Sing C, Eisen A, Foulkes WD, Rosen B (2014). Contralateral mastectomy and survival after breast cancer in carriers of BRCA1 and BRCA2 mutations: retrospective analysis. BMJ.

[10] Xiong Z, Yang L, Deng G, Huang X, Li X, Xie X, Wang J, Shuang Z, Wang X (2018). Patterns of occurrence and outcomes of contralateral breast cancer: analysis of SEER data. J Clin Med.

[11] Wong SM, Freedman RA, Sagara Y, Aydogan F, Barry WT, Golshan M (2017). Growing use of contralateral prophylactic mastectomy despite no improvement in long-term survival for invasive breast cancer. Ann Surg.

[12] Murphy JA, Milner TD, O'Donoghue JM (2013). Contralateral risk-reducing mastectomy in sporadic breast cancer. Lancet Oncol.

[13] Basu NN, Hodson J, Chatterjee S, Gandhi A, Wisely J, Harvey J, Highton L, Murphy J, Barnes N, Johnson R (2021). The Angelina Jolie effect: contralateral risk-reducing mastectomy trends in patients at increased risk of breast cancer. Sci Rep.

[14] Domchek SM (2019). Risk-reducing mastectomy in BRCA1 and BRCA2 mutation carriers: a complex discussion. JAMA.

[15] Giardiello D, Steyerberg EW, Hauptmann M, Adank MA, Akdeniz D, Blomqvist C, Bojesen SE, Bolla MK, Brinkhuis M, Chang-Claude J (2019). Prediction and clinical utility of a contralateral breast cancer risk model. Breast Cancer Res.

[16] Basu NN, Ross GL, Evans DG, Barr L (2015). The Manchester guidelines for contralateral risk-reducing mastectomy. World J Surg Oncol.

[17] Chowdhury M, Euhus D, Onega T, Biswas S, Choudhary PK (2017). A model for individualized risk prediction of contralateral breast cancer. Breast Cancer Res Treat.

[18] Chowdhury M, Euhus D, Arun B, Umbricht C, Biswas S, Choudhary P (2018). Validation of a personalized risk prediction model for contralateral breast cancer. Breast Cancer Res Treat.

[19] Weischer M, Nordestgaard BG, Pharoah P, Bolla MK, Nevanlinna H, Van't Veer LJ, Garcia-Closas M, Hopper JL, Hall P, Andrulis IL (2012). CHEK2*1100delC heterozygosity in women with breast cancer associated with early death, breast cancer-specific death, and increased risk of a second breast cancer. J Clin Oncol.

[20] Akdeniz D, Schmidt MK, Seynaeve CM, McCool D, Giardiello D, van den Broek AJ, Hauptmann M, Steyerberg EW, Hooning MJ (2019). Risk factors for metachronous contralateral breast cancer: a systematic review and meta-analysis. Breast.

[21] Robson ME, Reiner AS, Brooks JD, Concannon PJ, John EM, Mellemkjaer L, Bernstein L, Malone KE, Knight JA, Lynch CF (2017). Association of common genetic variants with contralateral breast cancer risk in the WECARE study. J Natl Cancer Inst.

[22] Fanale D, Incorvaia L, Filorizzo C, Bono M, Fiorino A, Calo V, Brando C, Corsini LR, Barraco N, Badalamenti G (2020). Detection of germline mutations in a cohort of 139 patients with bilateral breast cancer by multi-gene panel testing: impact of pathogenic variants in other genes beyond BRCA1/2. Cancers (Basel).

[23] Kramer I, Hooning MJ, Mavaddat N, Hauptmann M, Keeman R, Steyerberg EW, Giardiello D, Antoniou AC, Pharoah PDP, Canisius S (2020). Breast cancer polygenic risk score and contralateral breast cancer risk. Am J Hum Genet.

[24] Lakeman IMM, van den Broek AJ, Vos JAM, Barnes DR, Adlard J, Andrulis IL, Arason A, Arnold N, Arun BK, Balmana J (2021). The predictive ability of the 313 variant-based polygenic risk score for contralateral breast cancer risk prediction in women of European ancestry with a heterozygous BRCA1 or BRCA2 pathogenic variant. Genet Med.

[25] Mavaddat N, Michailidou K, Dennis J, Lush M, Fachal L, Lee A, Tyrer JP, Chen TH, Wang Q, Bolla MK (2019). Polygenic risk scores for prediction of breast cancer and breast cancer subtypes. Am J Hum Genet.

[26] Akdeniz D, Klaver MM, Smith CZA, Koppert LB, Hooning MJ (2020). The impact of lifestyle and reproductive factors on the risk of a second new primary cancer in the contralateral breast: a systematic review and meta-analysis. Cancer Causes Control.

[27] Pijpe A, Manders P, Brohet RM, Collee JM, Verhoef S, Vasen HF, Hoogerbrugge N, van Asperen CJ, Dommering C, Ausems MG (2010). Physical activity and the risk of breast cancer in BRCA1/2 mutation carriers. Breast Cancer Res Treat.

[28] Riegman PH, van Veen EB (2011). Biobanking residual tissues. Hum Genet.

[29] Foundation Federation of Dutch Medical Scientific Societies. Human tissue and medical research: code of conduct for responsible use. 2011.

[30] van den Broek AJ, Schmidt MK, van’t Veer LJ, Oldenburg HSA, Rutgers EJ, Russell NS, Smit V, Voogd AC, Koppert LB, Siesling S (2019). Prognostic impact of breast-conserving therapy versus mastectomy of BRCA1/2 mutation carriers compared with noncarriers in a consecutive series of young breast cancer patients. Ann Surg.

[31] Buuren S (2012). Flexible imputation of missing data.

[32] Resche-Rigon M, White IR, Bartlett JW, Peters SA, Thompson SG (2013). Group P-IS: Multiple imputation for handling systematically missing confounders in meta-analysis of individual participant data. Stat Med.

[33] Van Buuren S (2018). Flexible imputation of missing data.

[34] Geskus RB (2011). Cause-specific cumulative incidence estimation and the fine and gray model under both left truncation and right censoring. Biometrics.

[35] Schoenfeld DA (1983). Sample-size formula for the proportional-hazards regression model. Biometrics.

[36] Schmidt MK, Tollenaar RA, de Kemp SR, Broeks A, Cornelisse CJ, Smit VT, Peterse JL, van Leeuwen FE, Van't Veer LJ (2007). Breast cancer survival and tumor characteristics in premenopausal women carrying the CHEK2*1100delC germline mutation. J Clin Oncol.

[37] Schmidt MK, Hogervorst F, van Hien R, Cornelissen S, Broeks A, Adank MA, Meijers H, Waisfisz Q, Hollestelle A, Schutte M (2016). Age- and tumor subtype-specific breast cancer risk estimates for CHEK2*1100delC carriers. J Clin Oncol.

[38] Steyerberg EW, Harrell FE (2016). Prediction models need appropriate internal, internal-external, and external validation. J Clin Epidemiol.

[39] Austin PC, van Klaveren D, Vergouwe Y, Nieboer D, Lee DS, Steyerberg EW (2016). Geographic and temporal validity of prediction models: different approaches were useful to examine model performance. J Clin Epidemiol.

[40] Collins GS, Ogundimu EO, Altman DG (2016). Sample size considerations for the external validation of a multivariable prognostic model: a resampling study. Stat Med.

[41] Blanche P, Dartigues JF, Jacqmin-Gadda H (2013). Estimating and comparing time-dependent areas under receiver operating characteristic curves for censored event times with competing risks. Stat Med.

[42] Brentnall AR, Cuzick J (2020). Risk models for breast cancer and their validation. Stat Sci.

[43] Austin PC, Putter H, Giardiello D, van Klaveren D (2022). Graphical calibration curves and the integrated calibration index (ICI) for competing risk models. Diagn Progn Res.

[44] Collins GS, Reitsma JB, Altman DG, Moons KG (2015). Transparent reporting of a multivariable prediction model for individual prognosis or diagnosis (TRIPOD). Ann Intern Med.

[45] Vickers AJ, Elkin EB (2006). Decision curve analysis: a novel method for evaluating prediction models. Med Decis Mak.

[46] Kerr KF, Brown MD, Zhu K, Janes H (2016). Assessing the clinical impact of risk prediction models with decision curves: guidance for correct interpretation and appropriate use. J Clin Oncol.

[47] Vickers AJ, Cronin AM, Elkin EB, Gonen M (2008). Extensions to decision curve analysis, a novel method for evaluating diagnostic tests, prediction models and molecular markers. BMC Med Inform Decis Mak.

[48] Heemskerk-Gerritsen BA, Rookus MA, Aalfs CM, Ausems MG, Collee JM, Jansen L, Kets CM, Keymeulen KB, Koppert LB, Meijers-Heijboer HE (2015). Improved overall survival after contralateral risk-reducing mastectomy in BRCA1/2 mutation carriers with a history of unilateral breast cancer: a prospective analysis. Int J Cancer.

[49] Balmana J, Diez O, Rubio IT, Cardoso F, Group EGW (2011). BRCA in breast cancer: ESMO clinical practice guidelines. Ann Oncol.

[50] Rutgers EJT (2019). Is prophylactic mastectomy justified in women without BRCA mutation?. Breast.

[51] Giardiello D, Hauptmann M, Steyerberg EW, Adank MA, Akdeniz D, Blom JC, Blomqvist C, Bojesen SE, Bolla MK, Brinkhuis M (2020). Prediction of contralateral breast cancer: external validation of risk calculators in 20 international cohorts. Breast Cancer Res Treat.

[52] Antoniou AC, Pharoah PP, Smith P, Easton DF (2004). The BOADICEA model of genetic susceptibility to breast and ovarian cancer. Br J Cancer.

[53] Antoniou AC, Cunningham AP, Peto J, Evans DG, Lalloo F, Narod SA, Risch HA, Eyfjord JE, Hopper JL, Southey MC (2008). The BOADICEA model of genetic susceptibility to breast and ovarian cancers: updates and extensions. Br J Cancer.

[54] Lee AJ, Cunningham AP, Tischkowitz M, Simard J, Pharoah PD, Easton DF, Antoniou AC (2016). Incorporating truncating variants in PALB2, CHEK2, and ATM into the BOADICEA breast cancer risk model. Genet Med.

[55] Carver T, Hartley S, Lee A, Cunningham AP, Archer S, Babb de Villiers C, Roberts J, Ruston R, Walter FM, Tischkowitz M (2021). CanRisk Tool-A web interface for the prediction of breast and ovarian cancer risk and the likelihood of carrying genetic pathogenic variants. Cancer Epidemiol Biomarkers Prev.

[56] Kramer I, Schaapveld M, Oldenburg HSA, Sonke GS, McCool D, van Leeuwen FE, Van de Vijver KK, Russell NS, Linn SC, Siesling S (2019). The influence of adjuvant systemic regimens on contralateral breast cancer risk and receptor subtype. J Natl Cancer Inst.

[57] Witteveen A, Vliegen IM, Sonke GS, Klaase JM, Siesling S (2015). Personalisation of breast cancer follow-up: a time-dependent prognostic nomogram for the estimation of annual risk of locoregional recurrence in early breast cancer patients. Breast Cancer Res Treat.

[58] Volkel V, Hueting TA, Draeger T, van Maaren MC, de Munck L, Strobbe LJA, Sonke GS, Schmidt MK, van Hezewijk M, Groothuis-Oudshoorn CGM (2021). Improved risk estimation of locoregional recurrence, secondary contralateral tumors and distant metastases in early breast cancer: the INFLUENCE 2.0 model. Breast Cancer Res Treat.

[59] Nieboer D, Vergouwe Y, Ankerst DP, Roobol MJ, Steyerberg EW (2016). Improving prediction models with new markers: a comparison of updating strategies. BMC Med Res Methodol.

[60] Madley-Dowd P, Hughes R, Tilling K, Heron J (2019). The proportion of missing data should not be used to guide decisions on multiple imputation. J Clin Epidemiol.

[61] Collins GS, Altman DG (2012). Predicting the 10 year risk of cardiovascular disease in the United Kingdom: independent and external validation of an updated version of QRISK2. BMJ.

[62] Breast Cancer Association C, Dorling L, Carvalho S, Allen J, Gonzalez-Neira A, Luccarini C, Wahlstrom C, Pooley KA, Parsons MT, Fortuno C (2021). Breast cancer risk genes—association analysis in more than 113,000 women. N Engl J Med.

[63] Ho WK, Tan MM, Mavaddat N, Tai MC, Mariapun S, Li J, Ho PJ, Dennis J, Tyrer JP, Bolla MK (2020). European polygenic risk score for prediction of breast cancer shows similar performance in Asian women. Nat Commun.

[64] Evans DG, van Veen EM, Byers H, Roberts E, Howell A, Howell SJ, Harkness EF, Brentnall A, Cuzick J, Newman WG (2021). The importance of ethnicity: Are breast cancer polygenic risk scores ready for women who are not of White European origin?. Int J Cancer.

[65] Christodoulou E, Ma J, Collins GS, Steyerberg EW, Verbakel JY, Van Calster B (2019). A systematic review shows no performance benefit of machine learning over logistic regression for clinical prediction models. J Clin Epidemiol.

[66] Giardiello D, Antoniou AC, Mariani L, Easton DF, Steyerberg EW (2020). Letter to the editor: a response to Ming's study on machine learning techniques for personalized breast cancer risk prediction. Breast Cancer Res.

[67] Thompson D, Easton D (2004). The genetic epidemiology of breast cancer genes. J Mammary Gland Biol Neoplasia.

[68] Reiner AS, Sisti J, John EM, Lynch CF, Brooks JD, Mellemkjaer L, Boice JD, Knight JA, Concannon P, Capanu M (2018). Breast cancer family history and contralateral breast cancer risk in young women: an update from the women's environmental cancer and radiation epidemiology study. J Clin Oncol.

[69] Torkamani A, Wineinger NE, Topol EJ (2018). The personal and clinical utility of polygenic risk scores. Nat Rev Genet.

[70] Wald NJ, Old R (2019). The illusion of polygenic disease risk prediction. Genet Med.

[71] Knight JA, Blackmore KM, Fan J, Malone KE, John EM, Lynch CF, Vachon CM, Bernstein L, Brooks JD, Reiner AS (2018). The association of mammographic density with risk of contralateral breast cancer and change in density with treatment in the WECARE study. Breast Cancer Res.

[72] Van Belle V, Van Calster B (2015). Visualizing risk prediction models. PLoS ONE.

[73] Bonnett LJ, Snell KIE, Collins GS, Riley RD (2019). Guide to presenting clinical prediction models for use in clinical settings. BMJ.

[74] PREDICTCBC 2.0. https://www.evidencio.com/models/show/2949

