# Correction: PredictCBC-2.0: a contralateral breast cancer risk prediction model developed and validated in ~ 200,000 patients

**DOI:** 10.1186/s13058-022-01579-z

**Published:** 2022-11-22

**Authors:** Daniele Giardiello, Maartje J. Hooning, Michael Hauptmann, Renske Keeman, B. A. M. Heemskerk-Gerritsen, Heiko Becher, Carl Blomqvist, Stig E. Bojesen, Manjeet K. Bolla, Nicola J. Camp, Kamila Czene, Peter Devilee, Diana M. Eccles, Peter A. Fasching, Jonine D. Figueroa, Henrik Flyger, Montserrat García-Closas, Christopher A. Haiman, Ute Hamann, John L. Hopper, Anna Jakubowska, Floor E. Leeuwen, Annika Lindblom, Jan Lubiński, Sara Margolin, Maria Elena Martinez, Heli Nevanlinna, Ines Nevelsteen, Saskia Pelders, Paul D. P. Pharoah, Sabine Siesling, Melissa C. Southey, Annemieke H. van der Hout, Liselotte P. van Hest, Jenny Chang-Claude, Per Hall, Douglas F. Easton, Ewout W. Steyerberg, Marjanka K. Schmidt

**Affiliations:** 1 Division of Molecular Pathology, The Netherlands Cancer Institute - Antoni Van Leeuwenhoek Hospital, Plesmanlaan 121, 1066 CX Amsterdam, The Netherlands; 2 Department of Biomedical Data Sciences, Leiden University Medical Center, Leiden, The Netherlands; 3 Institute of Biomedicine, EURAC Research Affiliated Institute of the University of Lübeck, Bolzano, Italy; 4 Department of Medical Oncology, Erasmus MC Cancer Institute, Rotterdam, The Netherlands; 5 Brandenburg Medical School, Institute of Biostatistics and Registry Research, Neuruppin, Germany; 6 Institute of Medical Biometry and Epidemiology, University Medical Center Hamburg-Eppendorf, Hamburg, Germany; 7 Department of Oncology, Helsinki University Hospital, University of Helsinki, Helsinki, Finland; 8 Department of Oncology, Örebro University Hospital, Örebro, Sweden; 9 Copenhagen General Population Study, Herlev and Gentofte Hospital, Copenhagen University Hospital, Herlev, Denmark; 10 Department of Clinical Biochemistry, Herlev and Gentofte Hospital, Copenhagen University Hospital, Herlev, Denmark; 11 Faculty of Health and Medical Sciences, University of Copenhagen, Copenhagen, Denmark; 12 Department of Public Health and Primary Care, Centre for Cancer Genetic Epidemiology, University of Cambridge, Cambridge, UK; 13 Department of Internal Medicine and Huntsman Cancer Institute, University of Utah, Salt Lake City, UT USA; 14 Department of Medical Epidemiology and Biostatistics, Karolinska Institutet, Stockholm, Sweden; 15 Department of Pathology, Leiden University Medical Center, Leiden, The Netherlands; 16 Department of Human Genetics, Leiden University Medical Center, Leiden, The Netherlands; 17 Faculty of Medicine, University of Southampton, Southampton, UK; 18 Division of Hematology and Oncology, Department of Medicine, David Geffen School of Medicine, University of California at Los Angeles, Los Angeles, CA USA; 19 Department of Gynecology and Obstetrics, Comprehensive Cancer Center Erlangen-EMN, University Hospital Erlangen, Friedrich-Alexander University Erlangen-Nuremberg (FAU), Erlangen, Germany; 20 Usher Institute of Population Health Sciences and Informatics, The University of Edinburgh, Edinburgh, UK; 21 Cancer Research UK Edinburgh Centre, The University of Edinburgh, Edinburgh, UK; 22 Division of Cancer Epidemiology and Genetics, Department of Health and Human Services, National Cancer Institute, National Institutes of Health, Bethesda, MD USA; 23 Department of Breast Surgery, Herlev and Gentofte Hospital, Copenhagen University Hospital, Herlev, Denmark; 24 Department of Preventive Medicine, Keck School of Medicine, University of Southern California, Los Angeles, CA USA; 25 Molecular Genetics of Breast Cancer, German Cancer Research Center (DKFZ), Heidelberg, Germany; 26 Melbourne School of Population and Global Health, Centre for Epidemiology and Biostatistics, The University of Melbourne, Melbourne, VIC Australia; 27 Department of Genetics and Pathology, Pomeranian Medical University, Szczecin, Poland; 28 Independent Laboratory of Molecular Biology and Genetic Diagnostics, Pomeranian Medical University, Szczecin, Poland; 29 Division of Psychosocial Research and Epidemiology, The Netherlands Cancer Institute - Antoni Van Leeuwenhoek Hospital, Amsterdam, The Netherlands; 30 Department of Molecular Medicine and Surgery, Karolinska Institutet, Stockholm, Sweden; 31 Department of Clinical Genetics, Karolinska University Hospital, Stockholm, Sweden; 32 Department of Oncology, Södersjukhuset, Stockholm, Sweden; 33 Department of Clinical Science and Education, Karolinska Institutet, Södersjukhuset, Stockholm, Sweden; 34 Moores Cancer Center, University of California San Diego, La Jolla, CA USA; 35 Herbert Wertheim School of Public Health and Human Longevity Science, University of California San Diego, La Jolla, CA USA; 36 Department of Obstetrics and Gynecology, Helsinki University Hospital, University of Helsinki, Helsinki, Finland; 37 Department of Oncology, Leuven Multidisciplinary Breast Center, Leuven Cancer Institute, University Hospitals Leuven, Louven, Belgium; 38 Department of Oncology, Centre for Cancer Genetic Epidemiology, University of Cambridge, Cambridge, UK; 39 Department of Research and Development, Netherlands Comprehensive Cancer Organisation (IKNL), Utrecht, The Netherlands; 40 Department of HealthTechnology and Services Research, Technical Medical Centre, University of Twente, Enschede, The Netherlands; 41 Precision Medicine, School of Clinical Sciences at Monash Health, Monash University, Clayton, VIC Australia; 42 Department of Clinical Pathology, The University of Melbourne, Melbourne, VIC Australia; 43 Cancer Epidemiology Division, Cancer Council Victoria, Melbourne, VIC Australia; 44 Department of Genetics, University Medical Center Groningen, University Groningen, Groningen, The Netherlands; 45 Clinical Genetics, Amsterdam UMC, Vrije Universiteit Amsterdam, Amsterdam, The Netherlands; 46 Division of Cancer Epidemiology, German Cancer Research Center (DKFZ), Heidelberg, Germany; 47 Cancer Epidemiology Group, University Cancer Center Hamburg (UCCH), University Medical Center Hamburg-Eppendorf, Hamburg, Germany; 48 Department of Public Health, Erasmus MC Cancer Institute, Rotterdam, The Netherlands

## Correction to: Breast Cancer Research (2022) 24:69 10.1186/s13058-022-01567-3

Following publication of the original article [1], the authors flagged the following error in the '3. Formula to estimate the contralateral breast cancer risk using PredictCBC-2.0A' section of additional file 1: ‘+ 0.065 × I[Radiotherapy to the breast = yes]’ had been written in place of ‘− 0.065 × I[Radiotherapy to the breast = yes]‘.

The file has since been corrected. The authors thank you for reading and apologize for any inconvenience caused.
