# Supplementary material for: PredictCBC-2.0: a contralateral breast cancer risk prediction model developed and validated in ~ 200,000 patients
Source: Breast Cancer Res. 2022 Oct 21;24:69. doi: 10.1186/s13058-022-01567-3 (PMC9585761; doi:10.1186/s13058-022-01567-3)
Supplement: Supplementary file 1 — Additional file 1. Supplementary methods also including the following tables and figures Table S2. List of BCAC studies (including ABCS source) with the corresponding country and geographic area. Table S4: Clinical utility of the 5-year contralateral breast cancer risk prediction models (PredictCBC-1A with PredictCBC-2.0A and PredictCBC-1B with PredictCBC-2.0B). Figure S1. Visual assessment of calibration through calibration plots in the internal–external cross-validation at 5 years for the PredictCBC-2.0A model. Figure S2. Visual assessment of calibration through calibration plots in the internal–external cross-validation at 10 years for the PredictCBC-2.0A model. Figure S3. Visual assessment of calibration through calibration plots in the internal–external cross-validation at 5 years for the PredictCBC-2.0B model. Figure S4. Visual assessment of calibration through calibration plots in the internal–external cross-validation at 10 years for the PredictCBC-2.0B model. Figure S5. Density distribution of 5-year predicted contralateral breast cancer using PredictCBC-2.0 models. Figure S6. Decision curve analysis at 5 years for the contralateral breast cancer risk models (PredictCBC and PredictCBC-2.0) including BRCA mutation information. [file 13058_2022_1567_MOESM1_ESM.docx]

**Supplementary Materials**

Daniele Giardiello^1, 2, 3^, Maartje J. Hooning^4^, Michael Hauptmann^5^, Renske Keeman^1^, B. A. M. Heemskerk-Gerritsen^4^, Heiko Becher^6^, Carl Blomqvist^7, 8^, Stig E. Bojesen^9, 10, 11,^ Manjeet K. Bolla^12^, Nicola J. Camp^13^, Kamila Czene^14^, Peter Devilee^15, 16^, Diana M. Eccles^17^, Peter A. Fasching^18, 19^, Jonine D. Figueroa^20, 21, 22^, Henrik Flyger^23^, Montserrat García-Closas^22^, Christopher A. Haiman^24^, Ute Hamann^25^, John L. Hopper^26^, Anna Jakubowska^27, 28^, Floor E. Leeuwen^29^, Annika Lindblom^30, 31^, Jan Lubiński^27^, Sara Margolin^32, 33^, Maria Elena Martinez^34, 35^, Heli Nevanlinna^36^, Ines Nevelsteen^37^, Saskia Pelders^4^, Paul D.P. Pharoah^12, 38^, Sabine Siesling^39, 40^, Melissa C. Southey^41, 42, 43^, Annemieke H. van der Hout^44^, Liselotte P. van Hest^45^, Jenny Chang-Claude^46, 47^, Per Hall^14, 32^, Douglas F. Easton^12, 38^, Ewout W. Steyerberg^2, 48^, Marjanka K. Schmidt^1, 29^

^1^ The Netherlands Cancer Institute - Antoni van Leeuwenhoek Hospital, Division of Molecular Pathology, Amsterdam, The Netherlands.

^2^ Leiden University Medical Center, Department of Biomedical Data Sciences, Leiden, The Netherlands.

^3^ EURAC Research affiliated Institute of the University of Lübeck, Institute of Biomedicine, Bolzano/Bozen, Italy.

^4^ Erasmus MC Cancer Institute, Department of Medical Oncology, Rotterdam, The Netherlands.

^5^ Brandenburg Medical School, Institute of Biostatistics and Registry Research, Neuruppin, Germany.

^6^ University Medical Center Hamburg-Eppendorf, Institute of Medical Biometry and Epidemiology, Hamburg, Germany.

^7^ University of Helsinki, Department of Oncology, Helsinki University Hospital, Helsinki, Finland.

^8^ Örebro University Hospital, Department of Oncology, Örebro, Sweden.

^9^ Copenhagen University Hospital, Copenhagen General Population Study, Herlev and Gentofte Hospital, Herlev, Denmark.

^10^ Copenhagen University Hospital, Department of Clinical Biochemistry, Herlev and Gentofte Hospital, Herlev, Denmark.

^11^ University of Copenhagen, Faculty of Health and Medical Sciences, Copenhagen, Denmark.

^12^ University of Cambridge, Centre for Cancer Genetic Epidemiology, Department of Public Health and Primary Care, Cambridge, UK.

^13^ University of Utah, Department of Internal Medicine and Huntsman Cancer Institute, Salt Lake City, UT, USA.

^14^ Karolinska Institutet, Department of Medical Epidemiology and Biostatistics, Stockholm, Sweden.

^15^ Leiden University Medical Center, Department of Pathology, Leiden, The Netherlands.

^16^ Leiden University Medical Center, Department of Human Genetics, Leiden, The Netherlands.

^17^ University of Southampton, Faculty of Medicine, Southampton, UK.

^18^ University of California at Los Angeles, David Geffen School of Medicine, Department of Medicine Division of Hematology and Oncology, Los Angeles, CA, USA.

^19^ Comprehensive Cancer Center Erlangen-EMN, University Hospital Erlangen, Friedrich-Alexander University Erlangen-Nuremberg (FAU), Department of Gynecology and Obstetrics, Erlangen, Germany.

^20^ The University of Edinburgh, Usher Institute of Population Health Sciences and Informatics, Edinburgh, UK.

^21^ The University of Edinburgh, Cancer Research UK Edinburgh Centre, Edinburgh, UK.

^22^ National Cancer Institute, National Institutes of Health, Department of Health and Human Services, Division of Cancer Epidemiology and Genetics, Bethesda, MD, USA.

^23^ Copenhagen University Hospital, Department of Breast Surgery, Herlev and Gentofte Hospital, Herlev, Denmark.

^24^ University of Southern California, Department of Preventive Medicine, Keck School of Medicine, Los Angeles, CA, USA.

^25^ German Cancer Research Center (DKFZ), Molecular Genetics of Breast Cancer, Heidelberg, Germany.

^26^ The University of Melbourne, Centre for Epidemiology and Biostatistics, Melbourne School of Population and Global Health, Melbourne, Victoria, Australia.

^27^ Pomeranian Medical University, Department of Genetics and Pathology, Szczecin, Poland.

^28^ Pomeranian Medical University, Independent Laboratory of Molecular Biology and Genetic Diagnostics, Szczecin, Poland.

^29^ The Netherlands Cancer Institute - Antoni van Leeuwenhoek hospital, Division of Psychosocial Research and Epidemiology, Amsterdam, The Netherlands.

^30^ Karolinska Institutet, Department of Molecular Medicine and Surgery, Stockholm, Sweden.

^31^ Karolinska University Hospital, Department of Clinical Genetics, Stockholm, Sweden.

^32^ Södersjukhuset, Department of Oncology, Stockholm, Sweden.

^33^ Karolinska Institutet, Department of Clinical Science and Education, Södersjukhuset, Stockholm, Sweden.

^34^ University of California San Diego, Moores Cancer Center, La Jolla, CA, USA.

^35^ University of California San Diego, Herbert Wertheim School of Public Health and Human Longevity Science, La Jolla, CA, USA.

^36^ University of Helsinki, Department of Obstetrics and Gynecology, Helsinki University Hospital, Helsinki, Finland.

^37^ Leuven Cancer Institute, University Hospitals Leuven, Leuven Multidisciplinary Breast Center, Department of Oncology, Leuven, Belgium.

^38^ University of Cambridge, Centre for Cancer Genetic Epidemiology, Department of Oncology, Cambridge, UK.

^39^ Netherlands Comprehensive Cancer Organisation (IKNL), Department of Research and Development, Utrecht, The Netherlands.

^40^ Technical Medical Centre, University of Twente, Department of HealthTechnology and Services Research, Enschede, The Netherlands.

^41^ Monash University, Precision Medicine, School of Clinical Sciences at Monash Health, Clayton, Victoria, Australia.

^42^ The University of Melbourne, Department of Clinical Pathology, Melbourne, Victoria, Australia.

^43^ Cancer Council Victoria, Cancer Epidemiology Division, Melbourne, Victoria, Australia.

^44^ University Medical Center Groningen, University Groningen, Department of Genetics, Groningen, The Netherlands.

^45^ Amsterdam UMC, Vrije Universiteit Amsterdam, Clinical Genetics, Amsterdam, The Netherlands.

^46^ German Cancer Research Center (DKFZ), Division of Cancer Epidemiology, Heidelberg, Germany.

^47^ University Medical Center Hamburg-Eppendorf, Cancer Epidemiology Group, University Cancer Center Hamburg (UCCH), Hamburg, Germany.

^48^ Erasmus MC Cancer Institute, Department of Public Health, Rotterdam, The Netherlands.

Contents

[Supplementary Methods 5](#_Toc98147070)

[*1. Data and patient selection* 5](#_Toc98147071)

[*2. Multiple imputation of missing values* 7](#_Toc98147072)

[*3. Formula to estimate the contralateral breast cancer risk using PredictCBC-2.0A* 8](#_Toc98147073)

[*4. Formula to estimate the contralateral breast cancer risk in using PredictCBC-2.0B* 9](#_Toc98147074)

[*References* 10](#_Toc98147075)

[Supplementary Figures 17](#_Toc98147076)

# Supplementary Methods

## *1. Data and patient selection*

For this study we used data from six main sources available from national and international collaborations including nationwide registry data, as well as hospital-based studies with more detailed information on relevant prediction factors[1-5]. Briefly, the six main sources were: (1) The Breast Cancer Association Consortium (BCAC), which is an international consortium of 106 studies comprising 186,594 patients (data version August 2019) with a primary breast cancer (BC) diagnosed between 1939 and 2018[1]. In our previous study, 16 studies were selected to develop PredictCBC models. In this study, two studies were additionally included in the dataset to develop PredictCBC-2.0 models[6]; (2) The Amsterdam Breast Cancer Study (ABCS) containing 2,763 patients diagnosed with a first BC at the Netherlands Cancer Institute – Antoni van Leeuwenhoek (NKI-AVL) hospital in Amsterdam from 2003 to 2013[2]; (3) The Breast Cancer Outcome Study of Mutation carriers (BOSOM), which is a Dutch consecutive series of 7,105 patients with invasive BC treated for their primary BC in ten centers throughout the Netherlands between 1970 and 2003; in this study 94% of patients were genotyped for *BRCA1/2* germline mutations[3]; (4) The Erasmus Medical Center (EMC) study including patients diagnosed with BC between 1989 and 2013 who were treated at the EMC in Rotterdam; for this study, complete follow-up was obtained for 3,483 patients who had been diagnosed between 2000 and 2009;(5) The Netherlands Cancer Registry (NCR), which is an ongoing nationwide population-based data registry of all newly diagnosed cancer patients in the Netherlands since 1989[4]. We included patients diagnosed between 2003 and 2015, a period for which sufficient follow-up and receptor status information were available[4, 5];(6) Hereditary Breast and Ovarian cancer study, the Netherlands (HEBON) study is an ongoing nationwide Dutch study among members of *BRCA1/2* families in the Netherlands, including 16,617 BC patients diagnosed between 1953 and 2017[7]. The general design includes a retrospective cohort because the *BRCA1/2* DNA test was available from 1995, with a prospective follow-up. *BRCA1/2* families were identified through ten centers (nine Clinical Genetic Centers/Family Cancer Clinics and the Foundation for the Detection of Hereditary Tumors).. Data were harmonized by recoding each of the main datasets by the responsible data managers according to a standardized data dictionary. We performed checks for data consistency and validity centrally.

We extracted the following information: *BRCA1/2* germline mutation, family history (first degree) of primary BC, *CHEK2* c.1100delC, polygenic risk score (PRS) (derived from BCAC), body mass index (BMI), parity and regarding primary BC diagnosis: age, nodal status, size, grade, morphology, estrogen-receptor (ER) status, progesterone-receptor (PR), human epidermal growth factor receptor 2 (HER2) status, administration of adjuvant or neoadjuvant chemotherapy, adjuvant endocrine therapy, adjuvant trastuzumab therapy, radiotherapy[2, 8, 9]. We excluded PR status and TNM stage of the primary BC due to collinearity with ER status and the size of the primary tumor, respectively. In current clinical practice, only patients with ER-positive tumors receive endocrine therapy and only patients with HER2-positive tumors receive trastuzumab; these co-occurrences were considered in the model by using composite categorical variables. A description of the studies included in the analyses is provided in **Supplementary Table 1**. Follow-up started three months after invasive first primary BC diagnosis, to exclude synchronous contralateral breast cancer (CBC), and ended at date of CBC, distant metastasis (but not loco-regional relapse), CPM, or last date of follow-up (due to death, being lost to follow-up, or end of study), whichever occurred first. We considered that after loco-regional relapse, a woman would be still at risk for CBC as treatment for loco-regional relapse would not affect CBC unless adjuvant systemic treatment was given. Distant metastasis was considered as a competing risk because most of the patients receive systemic therapies after developing distant metastasis.

Age at first primary BC seemed to have a non-linear relation with CBC. Using splines, we observed that CBC risk increased with age till around 60 years old and declined afterwards. Therefore, we used a linear spline with a knot at 60 years in the prediction model. The use of this linear spline was a good compromise to address the non-linear relation between CBC risk and age across the different baseline risks in all the studies, with different age distributions and selections (one study included only women aged under 50 years). Moreover, the observed non-linear relation resembled the shape of age-related BC incidence curves with an increased risk until menopausal age followed by a decrease (Clemmensen’s hook)[10].

## *2. Multiple imputation of missing values*

The percentage of missing values across the predictors varied between 3.2% and 84% for morphology of first primary BC and *BRCA* mutation, respectively. In the individual patient data (IPD), both sporadic and systematic missing may occur. The former are missing values within a study, the latter are values missing for all individuals within a particular study[11-13].

For our analyses, we used five imputed datasets based on the multiple imputation chained equations (MICE) using 50 iterations. The visit sequence of the variables was in ascending order of the number of missing values. This technique improves the accuracy and the statistical power assuming missing is at random (MAR). In the imputation procedure, we also used the year of first primary BC diagnosis since this information provides a better correlation structure among covariates used as predictors in the imputation model. Since there were systematic missing data, we used the imputation model based on the stratified multiple imputation strategy (SMI). In this approach, the variable identifying the study was used as covariate to improve substantially the imputation especially for the systematic missing predictors that might occur in the IPD from multiple studies[13]. Continuous, binary, and multiple categorical variables were imputed using predictive mean matching, binary and multinomial logistic regression, respectively. Time-to-event outcome defined as time to CBC, time to death, and time to distant metastasis were included in the imputation process through the Nelson-Aalen cumulative hazard estimator[14]. For every variable with missing data, every imputation model selects predictors based on correlation structure underlying the data. We recoded the variables chemotherapy and morphology after imputation. Information about neoadjuvant and adjuvant chemotherapy were separately imputed. Then, we created a chemotherapy variable by combining the variables for neoadjuvant and adjuvant chemotherapy in every imputed dataset. Morphology of primary tumor was imputed by keeping all original categories (‘Lobular’, ‘Ductal’, ‘Mixed (ductal and lobular)’ and ‘Other’). After multiple imputation, we created two categories ‘Lobular including mixed’ and ‘Ductal including other’ to mitigate possible overfitting due to the small numbers of patients with ‘Mixed’ and ‘Other’ categories. Since in current clinical practice, only estrogen receptor (ER) positive patients receive endocrine therapy and only human epidermal growth factor receptor 2 (HER2) positive patients receive trastuzumab, composite categorical factors of ER and endocrine therapy and of HER2 and trastuzumab therapy were considered in the model building. However, in our data, 1% of patients with 97 CBC events were coded as ER-negative treated with endocrine therapy and 0.1% of patients with 11 CBC events were coded as HER2-negative treated with trastuzumab therapy. In every imputed dataset, we recoded those patients as ER-positive treated with endocrine treatment and HER2-positive treated with trastuzumab since the largest proportion of patients (67%) were ER-positive treated with endocrine therapy and 60% were HER2-positive treated with trastuzumab in the complete data.

We used the R package mice (version 3.13.0) to impute our data and combine the estimates using Rubin’s rules.

## *3. Formula to estimate the contralateral breast cancer risk using PredictCBC-2.0A*

Our developed model is a subdistributional proportional hazard Fine and Gray model. The estimated cumulative incidence of CBC was estimated using the following formula:

$$F\left( t \right)=1-\left\{ \left[ S_{0}(t) \right]^{exp(\boldsymbol{LP})} \right\}$$

Where *t* is the time (in years) since primary BC, $F\left( t \right)$ is the cumulative incidence of CBC and $S_{0}(t)$ is the probability to survive beyond for baseline covariate values. To calculate the predicted CBC cumulative incidence, we used the event-free baseline probability of the Dutch Cancer Registry. The baseline survival estimates according to the model and time *t* are:

$$S_{0}\left( 5 \right)= 0.985$$

$$S_{0}\left( 10 \right)= 0.971$$

And

*Linear Predictor* *(LP)* =

– 0.303 + 0.003× Age – 0.031 × Age’ + 0.011 × BMI – 0.0812 × Parity + 0.157 × I[Family history = Yes] + 1.566 × I[*BRCA = BRCA1*] + 1.128 × I[*BRCA = BRCA2*] + 0.938 × I[*CHEK2* c.1100delC] + 0.398 × PRS-313 – 0.011 × I[Nodal status = positive] – 0.089 × I[Size of PBC = (2,5] *cm*] + 0.201 × I[Size of PBC = greater than 5 *cm*] + 0.186 × I[Morphology of PBC = lobular including mixed] – 0.069 × I[Grade of PBC = moderately differentiated] – 0.163 × I[Grade of PBC = poorly/undifferentiated]– 0.285 × I[Chemotherapy = yes] + 0.065 × I[Radiotherapy to the breast = yes] + 0.428 × I[ER-negative without endocrine therapy ] + 0.668 × I[ER-positive without endocrine therapy ]+ 0.203 × I[HER2-negative without trastuzumab] + 0.111 × I[HER2-positive without trastuzumab]

Where Age’ = max(Age – 60, 0), with age in years

## *4. Formula to estimate the contralateral breast cancer risk in using PredictCBC-2.0B*

The formula for the alternative model is reported below. Baseline survival estimates according to the model and time *t* are:

$$S_{0}\left( 5 \right)= 0.984$$

$$S_{0}\left( 10 \right)= 0.970$$

And

– 0.160 – 0.002 × Age – 0.029 × Age’ + 0.011 × BMI – 0.0728 × Parity + 0.304 × I[Family history = Yes] – 0.013× I[Nodal status = positive] + 0.011 × I[Size of PBC = (2,5] *cm*] + 0.198 × I[Size of PBC = greater than 5 *cm*] + 0.158 × I[Morphology of PBC = lobular including mixed] – 0.017 × I[Grade of PBC = moderately differentiated] – 0.055 × I[Grade of PBC = poorly/undifferentiated]– 0.293 × I[Chemotherapy = yes] – 0.055 × I[Radiotherapy to the breast = yes] + 0.578 × I[ER-negative without endocrine therapy ] + 0.661 × I[ER-positive without endocrine therapy ]+ 0.262 × I[HER2-negative without trastuzumab] + 0.133 × I[HER2-positive without trastuzumab]

Where Age’ = max(Age – 60, 0), with age in years

## *References*

1. Michailidou K, Lindstrom S, Dennis J, Beesley J, Hui S, Kar S, Lemacon A, Soucy P, Glubb D, Rostamianfar A *et al*: **Association analysis identifies 65 new breast cancer risk loci**. *Nature* 2017, **551**(7678):92-94.

2. Schmidt MK, Tollenaar RA, de Kemp SR, Broeks A, Cornelisse CJ, Smit VT, Peterse JL, van Leeuwen FE, Van't Veer LJ: **Breast cancer survival and tumor characteristics in premenopausal women carrying the CHEK2*1100delC germline mutation**. *J Clin Oncol* 2007, **25**(1):64-69.

3. Schmidt MK, van den Broek AJ, Tollenaar RA, Smit VT, Westenend PJ, Brinkhuis M, Oosterhuis WJ, Wesseling J, Janssen-Heijnen ML, Jobsen JJ *et al*: **Breast Cancer Survival of BRCA1/BRCA2 Mutation Carriers in a Hospital-Based Cohort of Young Women**. *J Natl Cancer Inst* 2017, **109**(8).

4. Font-Gonzalez A, Liu L, Voogd AC, Schmidt MK, Roukema JA, Coebergh JW, de Vries E, Soerjomataram I: **Inferior survival for young patients with contralateral compared to unilateral breast cancer: a nationwide population-based study in the Netherlands**. *Breast Cancer Res Treat* 2013, **139**(3):811-819.

5. Kramer I, Schaapveld M, Oldenburg HSA, Sonke GS, McCool D, Van Leeuwen FE, van de Vijver KK, Russell NS, Linn SC, Siesling S *et al*: **The influence of adjuvant systemic regimens on contralateral breast cancer risk and receptor subtype**. *J Natl Cancer Inst* In press.

6. Giardiello D, Steyerberg EW, Hauptmann M, Adank MA, Akdeniz D, Blomqvist C, Bojesen SE, Bolla MK, Brinkhuis M, Chang-Claude J *et al*: **Prediction and clinical utility of a contralateral breast cancer risk model**. *Breast Cancer Res* 2019, **21**(1):144.

7. Pijpe A, Manders P, Brohet RM, Collee JM, Verhoef S, Vasen HF, Hoogerbrugge N, van Asperen CJ, Dommering C, Ausems MG *et al*: **Physical activity and the risk of breast cancer in BRCA1/2 mutation carriers**. *Breast Cancer Res Treat* 2010, **120**(1):235-244.

8. Mavaddat N, Michailidou K, Dennis J, Lush M, Fachal L, Lee A, Tyrer JP, Chen TH, Wang Q, Bolla MK *et al*: **Polygenic Risk Scores for Prediction of Breast Cancer and Breast Cancer Subtypes**. *Am J Hum Genet* 2019, **104**(1):21-34.

9. Kramer I, Hooning MJ, Mavaddat N, Hauptmann M, Keeman R, Steyerberg EW, Giardiello D, Antoniou AC, Pharoah PDP, Canisius S *et al*: **Breast Cancer Polygenic Risk Score and Contralateral Breast Cancer Risk**. *Am J Hum Genet* 2020, **107**(5):837-848.

10. Bouchardy C, Usel M, Verkooijen HM, Fioretta G, Benhamou S, Neyroud-Caspar I, Schaffar R, Vlastos G, Wespi Y, Schafer P *et al*: **Changing pattern of age-specific breast cancer incidence in the Swiss canton of Geneva**. *Breast Cancer Res Treat* 2010, **120**(2):519-523.

11. Riley RD, Lambert PC, Abo-Zaid G: **Meta-analysis of individual participant data: rationale, conduct, and reporting**. *BMJ* 2010, **340**:c221.

12. Resche-Rigon M, White IR, Bartlett JW, Peters SA, Thompson SG, Group P-IS: **Multiple imputation for handling systematically missing confounders in meta-analysis of individual participant data**. *Stat Med* 2013, **32**(28):4890-4905.

13. Jolani S, Debray TP, Koffijberg H, van Buuren S, Moons KG: **Imputation of systematically missing predictors in an individual participant data meta-analysis: a generalized approach using MICE**. *Stat Med* 2015, **34**(11):1841-1863.

14. White IR, Royston P: **Imputing missing covariate values for the Cox model**. *Stat Med* 2009, **28**(15):1982-1998.

**Table S1**: see additional file 2: Table S1

**Table S2:** List of BCAC studies (including ABCS source) with the corresponding country and geographic area. For studies in which the number of contralateral breast cancer events was insufficient for external validation, the geographic area was used.

| **Study** | **Country** | **Geographic area or study** |
| --- | --- | --- |
| ABCS | Netherlands | Europe - Other |
| ABCFS | Australia | United States and Australia |
| BBCC | Germany | Europe - Other |
| BREOGAN | Spain | Europe - Other |
| CGPS | Denmark | Europe - Scandinavia |
| HEBCS | Finland | Europe - Scandinavia |
| KARBAC | Sweden | Europe - Scandinavia |
| KARMA | Sweden | Europe - Scandinavia |
| LMBC | Belgium | Europe - Other |
| MARIE | Germany | Europe - Other |
| MEC | United States | United States and Australia |
| ORIGO | Netherlands | Europe - Other |
| PBCS | Poland | Europe - Other |
| PKARMA | Sweden | Europe - Scandinavia |
| POSH | United Kingdom | Europe - United Kingdom |
| SEARCH | United Kingdom | Europe - United Kingdom |
| SKKDKFZS | Germany | Europe - Other |
| SZBCS | Poland | Europe - Other |
| UBCS | United States | United States and Australia |
|  | | |

**Table S3**: see Additional file 3: Table S3 (Patient and primary breast cancer characteristics per study).

**Table S4**: Clinical utility of the 5-year contralateral breast cancer risk prediction models (PredictCBC-1A with PredictCBC-2.0A and PredictCBC-1B with PredictCBC-2.0B). For PredictCBC versions 1A and 2.0A, at the same probability threshold, the net benefit is exemplified in *BRCA1/2* mutation carriers (for avoiding unnecessary CPM) and non-carriers (performing necessary CPM). For PredictCBC versions 1B and 2.0B, at the same probability threshold, the net benefit is exemplified in patients with family history (for avoiding unnecessary CPM) and patients without family history (performing necessary CPM).

| PredictCBC-1A and PredictCBC-2.0A | | | | | | | |
| --- | --- | --- | --- | --- | --- | --- | --- |
| Probability threshold p_t_ (%) | Unnecessary CPMs needed  to detect one  necessary CPM* | *BRCA1/2* mutation carriers | | | Non-carriers | | |
|  |  | Net benefit  versus  treat all patients with CPM (per 1000) | Avoided  unnecessary CPMs  per 1000 patients  using  PredictCBC-1A | Additional avoided unnecessary CPMs per 1000 patients using PredictCBC-2.0A | Net benefit  versus  treat none (per 1000) | Performed necessary CPMs per 1000 patients  using  PredictCBC-1A | Additional performed necessary CPMs per 1000 patients using PredictCBC-2.0A |
| 3 | 32.3 | 0.2 | 6.0 | 0.0 | 0.6 | 19.7 | 210.9 |
| 4 | 24.0 | 1.9 | 44.4 | 16.4 | No benefit | 0.0 | 129.4 |
| 5 | 19.0 | 3.4 | 64.1 | 66.7 | No benefit | 0.0 | 56.9 |
| 6 | 15.7 | 9.4 | 146.6 | 34.1 | No benefit | 0.0 | 0.0 |
| PredictCBC-1B and PredictCBC-2.0B | | | | | | | |
| Probability threshold p_t_ (%) | Unnecessary CPMs needed  to detect one  necessary CPM* | Family history | | | No family history | | |
|  |  | Net benefit  versus  treat all patients with CPM (per 1000) | Avoided  unnecessary CPMs  per 1000 patients  using  PredictCBC-1B | Additional avoided unnecessary CPMs per 1000 patients using PredictCBC-2.0B | Net benefit  versus  treat none (per 1000) | Performed necessary CPMs per 1000 patients  using  PredictCBC-1B | Additional performed necessary CPMs per 1000 patients using PredictCBC-2.0B |
| 2 | 49 | 2.3 | 115.1 | 0.0 | 3.4 | 168.1 | 0.0 |
| 2.5 | 39 | 5.7 | 200.4 | 0.0 | 1.8 | 70.1 | 0.0 |
| 3 | 32.3 | 3.6 | 258.3 | 0.0 | 0.6 | 19.9 | 0.3 |
| CPM: contralateral preventive mastectomy; * The number of unnecessary contralateral mastectomies needed to detect one necessary CPM is calculated by: (1-pt)/pt | | | | | | | |

# Supplementary Figures


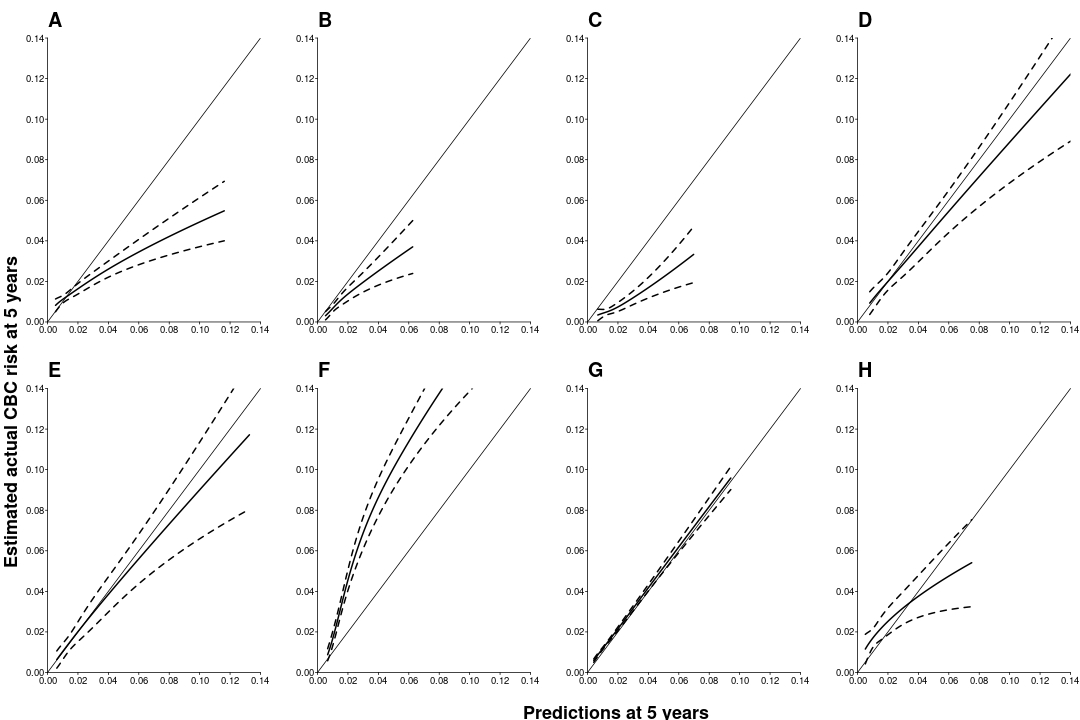


**Figure S1:** Visual assessment of calibration through calibration plots in the internal-external cross-validation at 5 years for the PredictCBC-2.0A model.

The x-axis represents the predicted cumulative incidence of contralateral breast cancer estimated by PredictCBC-2.0A model at 5 years and the y-axis the estimated actual contralateral breast cancer risk at 5 years. The black lines indicate the calibration of predicted values using an three-knot restricted cubic spline. Dashed black lines indicate the 95% confidence intervals. The dashed gray line indicates perfect overall calibration. Each panel indicates a validation in one of the datasets. Panel A: Europe – Other ; Panel B: Europe – Scandinavia; Panel C: Europe – UK ; Panel D: Netherlands – BOSOM; Panel E: Netherlands – EMC; Panel F: Netherlands – HEBON; Panel G: Netherlands – NCR; Panel H: US and Australia.


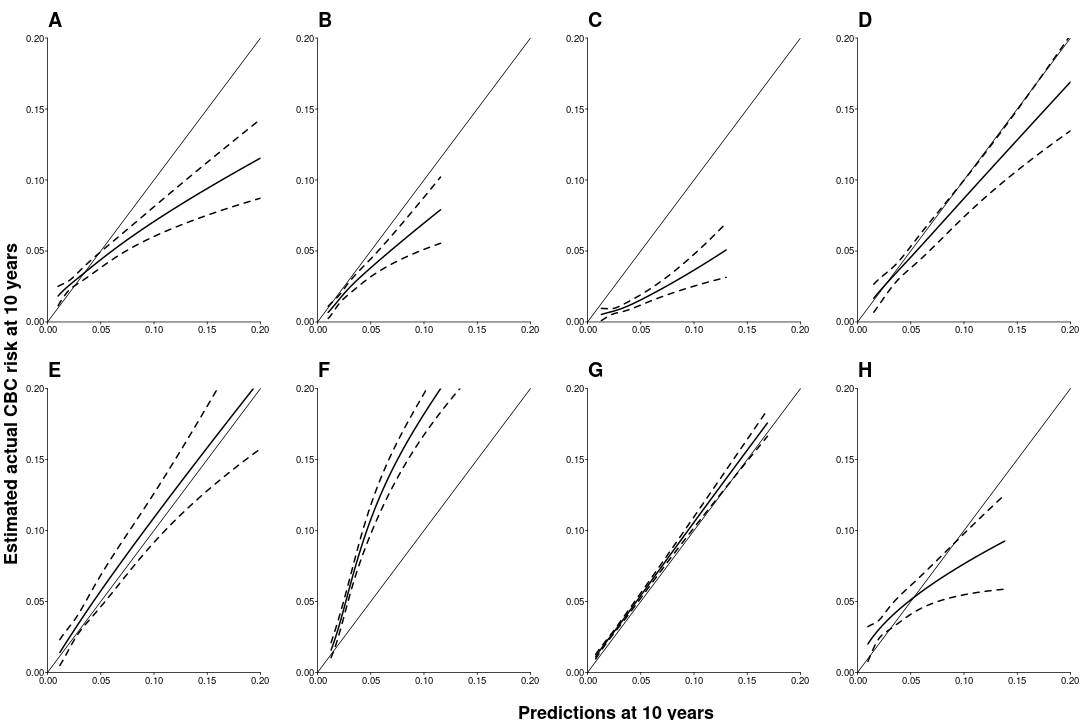


**Figure S2:** Visual assessment of calibration through calibration plots in the internal-external cross-validation at 10 years for the PredictCBC-2.0A model.

The x-axis represents the predicted cumulative incidence of contralateral breast cancer estimated by PredictCBC-2.0A model at 10 years and the y-axis the estimated actual contralateral breast cancer risk at 10 years. The black lines indicate the calibration of predicted values using a three-knot restricted cubic spline. Dashed black lines indicate the 95% confidence intervals. The dashed gray line indicates perfect overall calibration. Each panel indicates a validation in one of the datasets. Panel A: Europe – Other; Panel B: Europe – Scandinavia; Panel C: Europe – UK ; Panel D: Netherlands – BOSOM; Panel E: Netherlands – EMC; Panel F: Netherlands – HEBON; Panel G: Netherlands – NCR; Panel H: US and Australia.


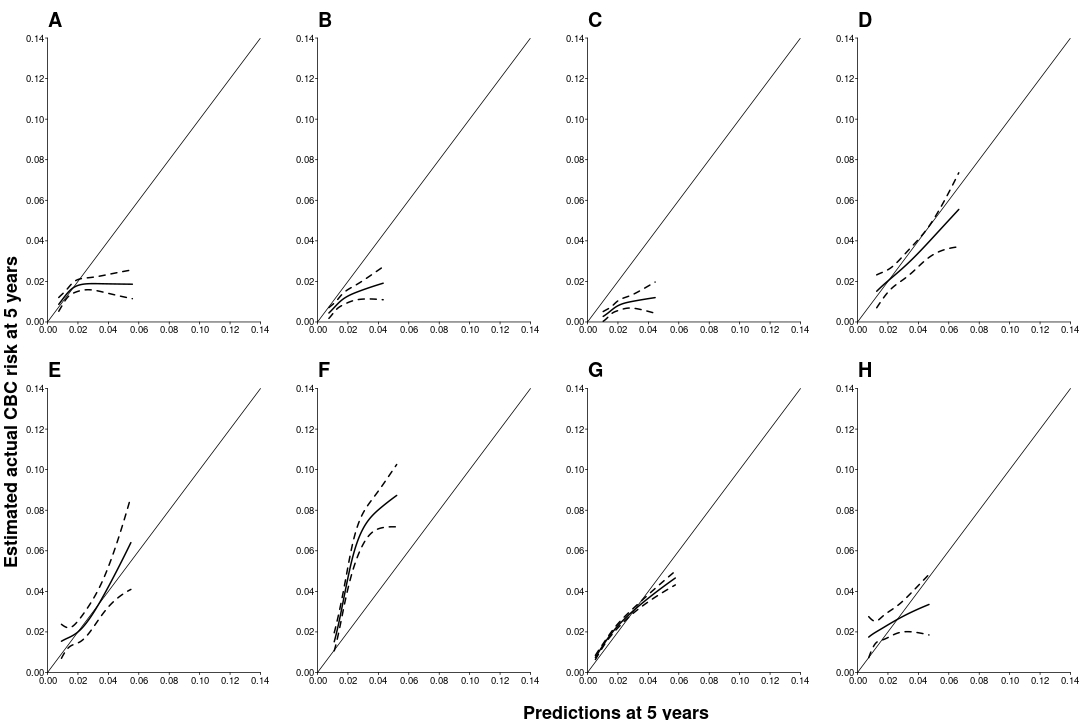


**Figure S3:** Visual assessment of calibration through calibration plots in the internal-external cross-validation at 5 years for the PredictCBC-2.0B model.

The x-axis represents the predicted cumulative incidence of contralateral breast cancer estimated by PredictCBC-2.0B model at 5 years and the y-axis the estimated actual contralateral breast cancer risk at 5 years. The black lines indicate the calibration of predicted values using a three-knot restricted cubic spline. Dashed black lines indicate the 95% confidence intervals. The dashed gray line indicates perfect overall calibration. Each panel indicates a validation in one of the datasets. Panel A: Europe – Other; Panel B: Europe – Scandinavia; Panel C: Europe – UK ; Panel D: Netherlands – BOSOM; Panel E: Netherlands – EMC; Panel F: Netherlands – HEBON; Panel G: Netherlands – NCR; Panel H: US and Australia.


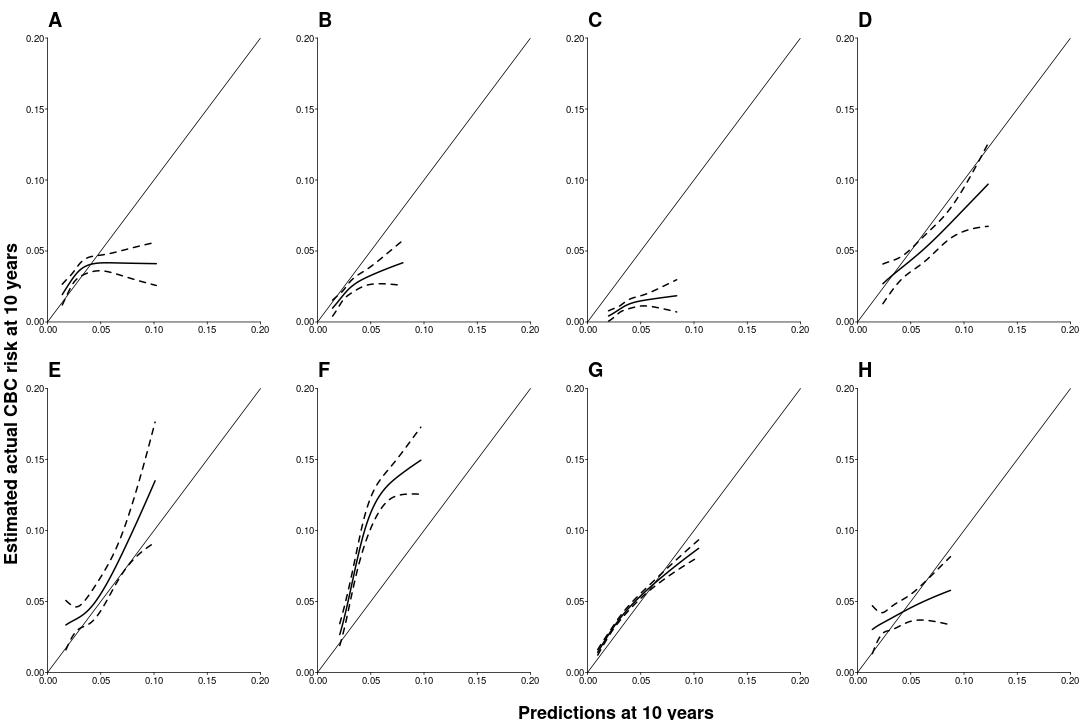


**Figure S4:** Visual assessment of calibration through calibration plots in the internal-external cross-validation at 10 years for the PredictCBC-2.0B model.

The x-axis represents the predicted cumulative incidence of contralateral breast cancer estimated by PredictCBC-2.0B model at 10 years and the y-axis the estimated actual contralateral breast cancer risk at 10 years. The black lines indicate the calibration of predicted values using an three-knot restricted cubic spline. Dashed black lines indicate the 95% confidence intervals. The dashed gray line indicates perfect overall calibration. Each panel indicates a validation in one of the datasets. Panel A: Europe – Other ; Panel B: Europe – Scandinavia; Panel C: Europe – UK ; Panel D: Netherlands – BOSOM; Panel E: Netherlands – EMC; Panel F: Netherlands – HEBON; Panel G: Netherlands – NCR; Panel H: US and Australia.


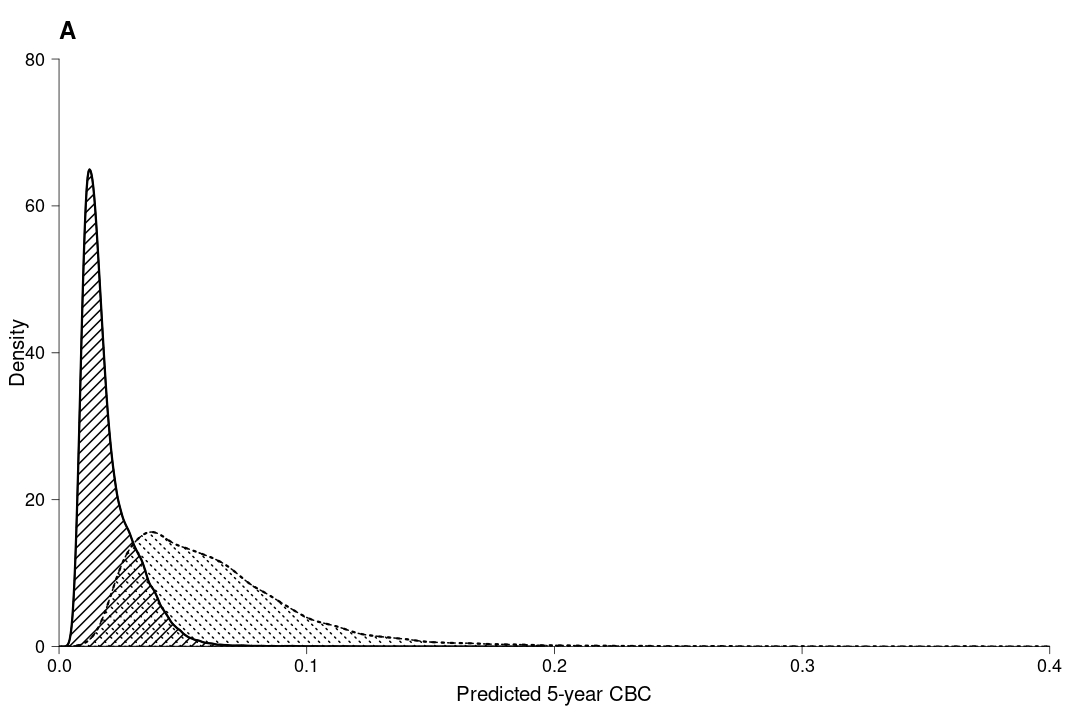

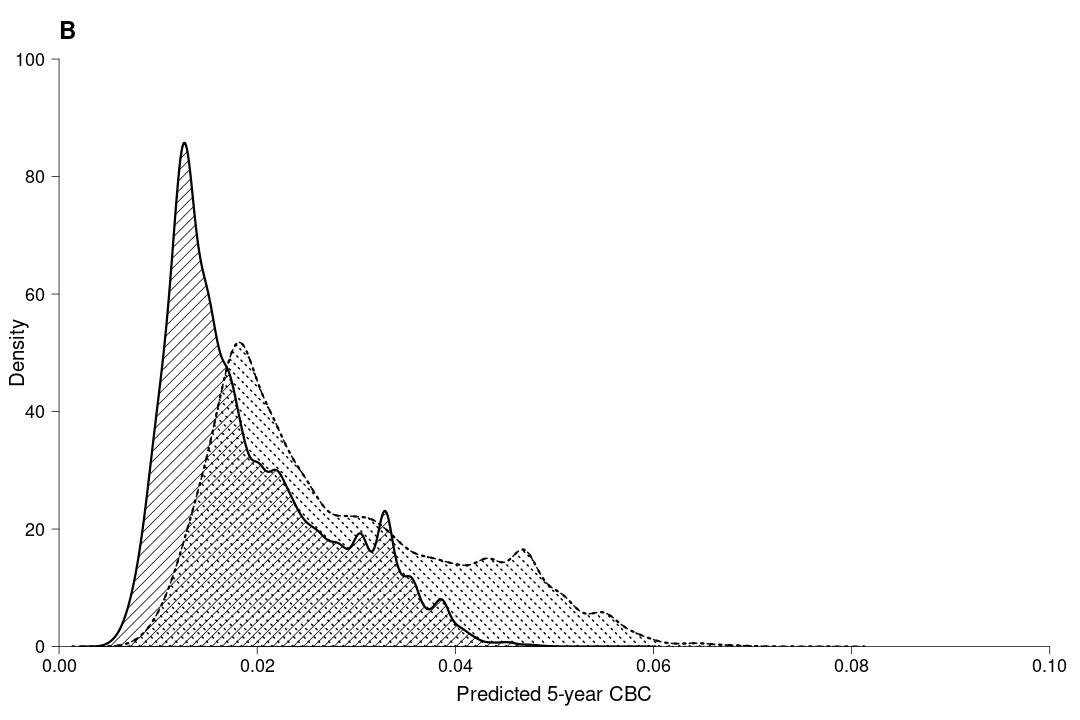


**Figure S5:** Density distribution of 5-year predicted contralateral breast cancer using PredictCBC-2.0 models. **a** Density distribution of 5-year predicted contralateral breast cancer absolute risk using PredictCBC-2.0A within non-carriers (area with black solid lines) and *BRCA1/2* mutation carriers (area with black dashed lines). **b** Density distribution of 5-year predicted contralateral breast cancer absolute risk using PredictCBC-2.0B within patients without (first degree) family history (area with black solid lines) and patients with (first degree) family history (area with black dashed lines).


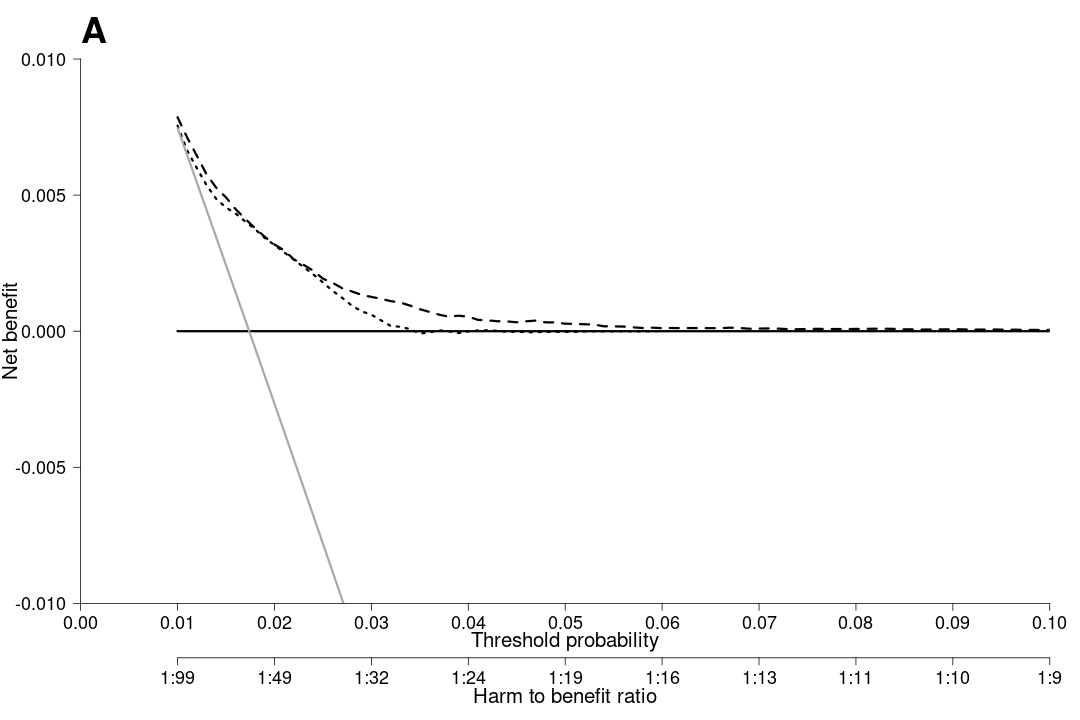

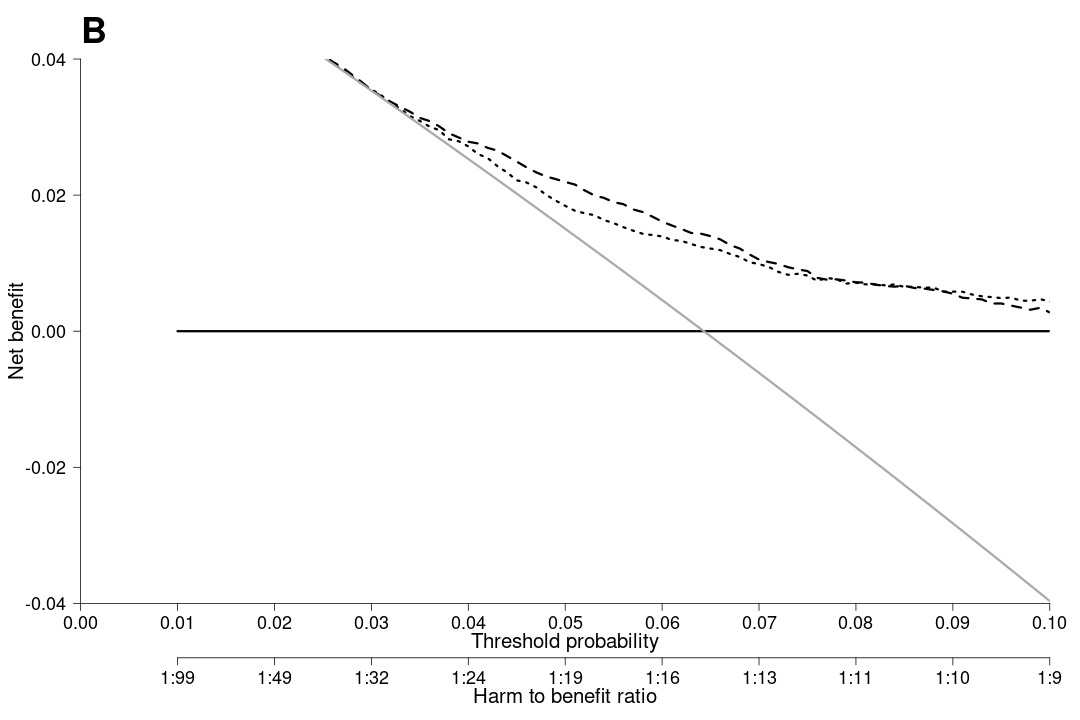

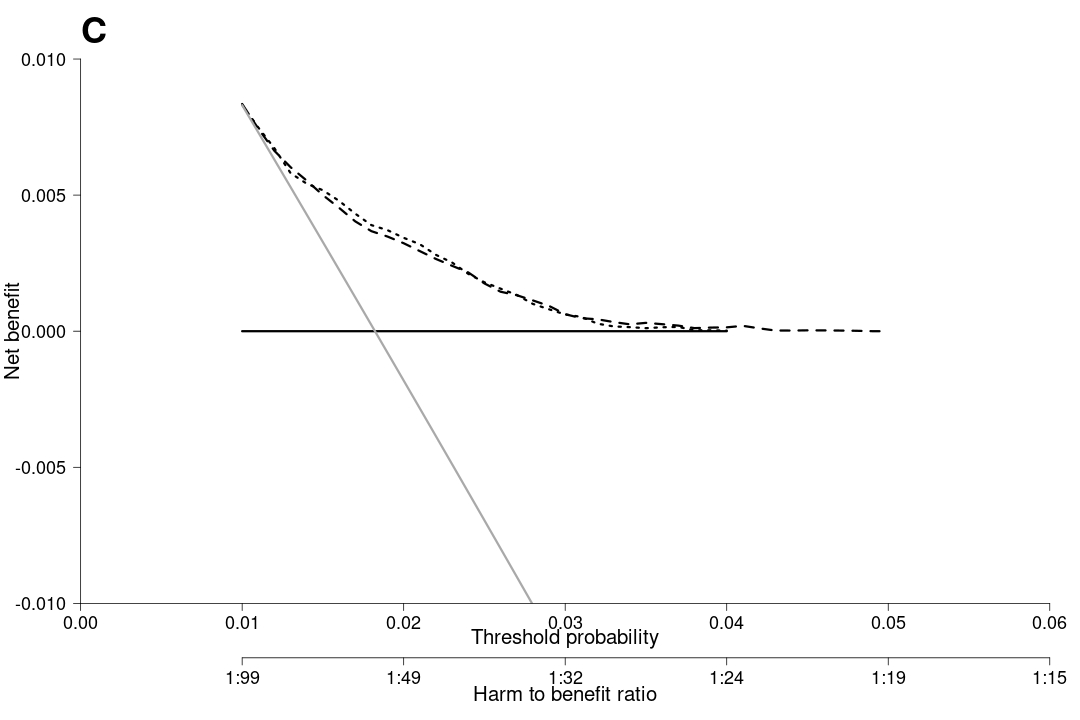

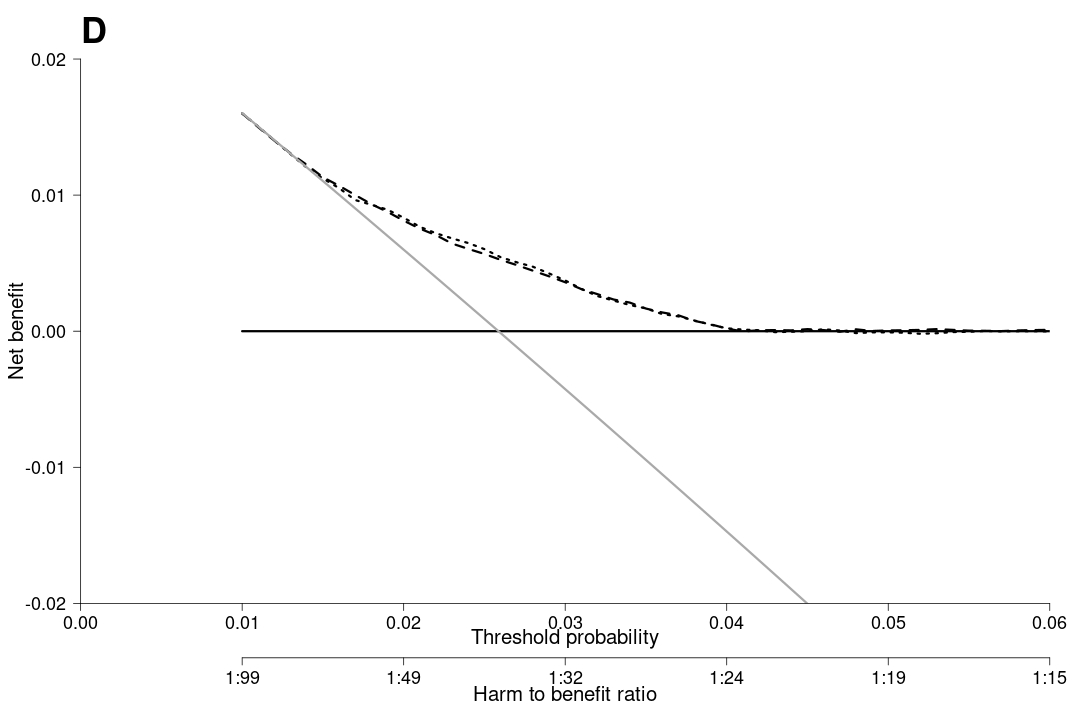


**Figure S6.** Decision curve analysis at 5 years for the contralateral breast cancer risk models (PredictCBC and PredictCBC-2.0) including *BRCA* mutation information. **a** The decision curve to determine the net benefit of the estimated 5-year predicted contralateral breast cancer (CBC) cumulative incidence for patients without a *BRCA1/2* gene mutation using PredictCBC-1A (dotted black line) and PredictCBC-2.0A (dashed black line) compared to not treating any patients with contralateral preventive mastectomy (CPM) (black solid line). **b** The decision curve to determine the net benefit of the estimated 5-year predicted CBC cumulative incidence for *BRCA1/2* mutation carriers using PredictCBC-1A (dotted black line), PredictCBC-2.0A (dashed black line) versus treating (or at least counseling) all patients (gray solid line). **c** The decision curve to determine the net benefit of the estimated 5-year predicted CBC cumulative incidence for patients without (first-degree) family history using PredictCBC-1B (dotted black line), PredictCBC-2.0B (dashed black line) compared to not treating any patients with CPM (black solid line). **d** The decision curve to determine the net benefit of the estimated 5-year predicted CBC cumulative incidence for patients with (first-degree) family history using PredictCBC-1B (dotted black line), PredictCBC-2.0B (dashed black line) versus treating (or at least counseling) all patients (gray solid line). The y-axis measures net benefit, which is calculated by summing the benefits (true positives, i.e., patients with a CBC who needed a CPM) and subtracting the harms (false positives, i.e., patients with CPM who do not need it). The latter are weighted by a factor related to the relative harm of a non-prevented CBC versus an unnecessary CPM. The factor is derived from the threshold probability to develop a CBC at 10 years at which a patient would opt for CPM (e.g., 5%). The x-axis represents the threshold probability. Using a threshold probability of 5% implicitly means that CPM in 20 patients of whom one would develop a CBC if untreated is acceptable (19 unnecessary CPMs, harm to benefit ratio 1:19)
